# Integrating field- and remote sensing data to perceive species heterogeneity across a climate gradient

**DOI:** 10.1038/s41598-023-50812-y

**Published:** 2024-01-02

**Authors:** Amrita N. Chaurasia, Reshma M. Parmar, Maulik G. Dave, N. S. R. Krishnayya

**Affiliations:** Ecology Laboratory, Department of Botany, The Maharaja Sayajirao University of Baroda, Vadodara, Gujarat 390002 India

**Keywords:** Ecology, Plant sciences

## Abstract

Tropical forests exhibit significant diversity and heterogeneity in species distribution. Some tree species spread abundantly, impacting the functional aspects of communities. Understanding how these facets respond to climate change is crucial. Field data from four protected areas (PAs) were combined with high-resolution Airborne Visible/InfraRed Imaging Spectrometer-Next Generation (AVIRIS-NG) datasets to extract large-scale plot data of abundant species and their functional traits. A supervised component generalized linear regression (SCGLR) model was used to correlate climate components with the distribution of abundant species across PAs. The recorded rainfall gradient influenced the proportion of PA-specific species in the observed species assemblages. Community weighted means (CWMs) of biochemical traits showed better correlation values (0.85–0.87) between observed and predicted values compared to biophysical traits (0.52–0.79). The model-based projection revealed distinct distribution responses of each abundant species to the climate gradient. Functional diversity and functional traits maps highlighted the interplay between species heterogeneity and climate. The appearance dynamics of abundant species in dark diversity across PAs demonstrated their assortment strategy in response to the climate gradient. These observations can significantly aid in the ecological management of PAs exposed to climate dynamics.

## Introduction

Tropical forests show high species diversity and heterogeneity. Some of the tree species become more abundant and have sizable contribution to the forest ecosystem processes. Dispersal and recruitment limitations^1^ along with the adaptive nature of a species to abiotic conditions^2^ impact species heterogeneity. It is important to assess how species assort themselves to bring in compositional heterogeneity^3^ more suitable to local climatic conditions. Examining community dynamics across a climate gradient is vital to project the impact of climate change^4^ on the existing forest covers. Species response to the surrounding abiotic conditions is dynamic and is visible in their functional trait amplitudes. Better inputs at a larger scale are required for models to study how forest tree cover responds to climate over species assortment and their trait characteristics^5^. Current available details on tree species, their classification is limited for Asia^6^. Assessing the importance of abundant species to the functioning of tree communities over a climate gradient has an immense importance^7^ as regions in Asia have diverse climate and are growing rapidly.

Generating extensive large-scale data on tree covers is a herculean task. It is imperative to build a better understanding of the ecological processes of larger regions from small-scale studies^8^. Combining small-scale field observations with remote sensing data to monitor biodiversity at larger scales is advantageous^9^. Critical inputs such as community composition and primary productivity, can be extracted from remote sensing data^9^. The impact of environmental variability on species response and heterogeneity amongst functional groups provide crucial information on how tree species respond to climate change^10^. Elucidating remote sensing data to analyze ecological processes^11^ such as dynamics in species assortment becomes important for the better assessment of forest covers. Different studies have been conducted syncing field-based ecological observations on essential biodiversity variables with remote sensing data^9^ for a finer comprehension of forest cover dynamics.

Other than assisting in wall-to-wall monitoring of biophysical features of forest tree covers^12^, spectral reflectance data can be facilitating the extraction of different vegetation indices that can be ideal proxies for vital canopy functional traits. Conventional measurements are relatively unrealistic to generate trait characteristics at larger scales^13^. Limited information is available on trait variability among species over climate gradient^14^. Few of these remote sensing based indices such as Chlorophyll/Carotenoid Index (CCI)^15^, Near-infrared reflectance of vegetation multiplied by incoming sunlight (NIRvP)^16,17^ are very useful in estimating light harvesting activities of forest covers^18^. NIRvP can effectively capture variability in gross primary productivity across time scales^19^ and can be a better proxy over sun-induced chlorophyll fluorescence in capturing the canopy photosynthesis and phenology of deciduous and evergreen vegetation over large spatial scales^20^. The Normalized Difference Water Index (NDWI)^21^ is another useful index having the potential to estimate water content at the canopy scale^22,23^. These indices can be effectively extracted from high-resolution remote sensing data to make them very useful in bringing the physiological response of species to climate gradient over larger spatial scales. Combining these with community heterogeneity highlight functional diversity of tree covers assisting further in perceiving forests’ response to climate change.

Abundant species of a forest may not be spreading uniformly across the area. Few species could be absent in a local community but could be seen frequently with the other species present in that local community, and these absent species collectively are referred to as dark diversity (DD)^2^. It can be obtained by estimations from field study^24^ and can be tried to extract from the species classification maps. Listing up of species falling in DD can assist in evaluating the ability of the species to spread across a climate gradient.

**Figure 1:**
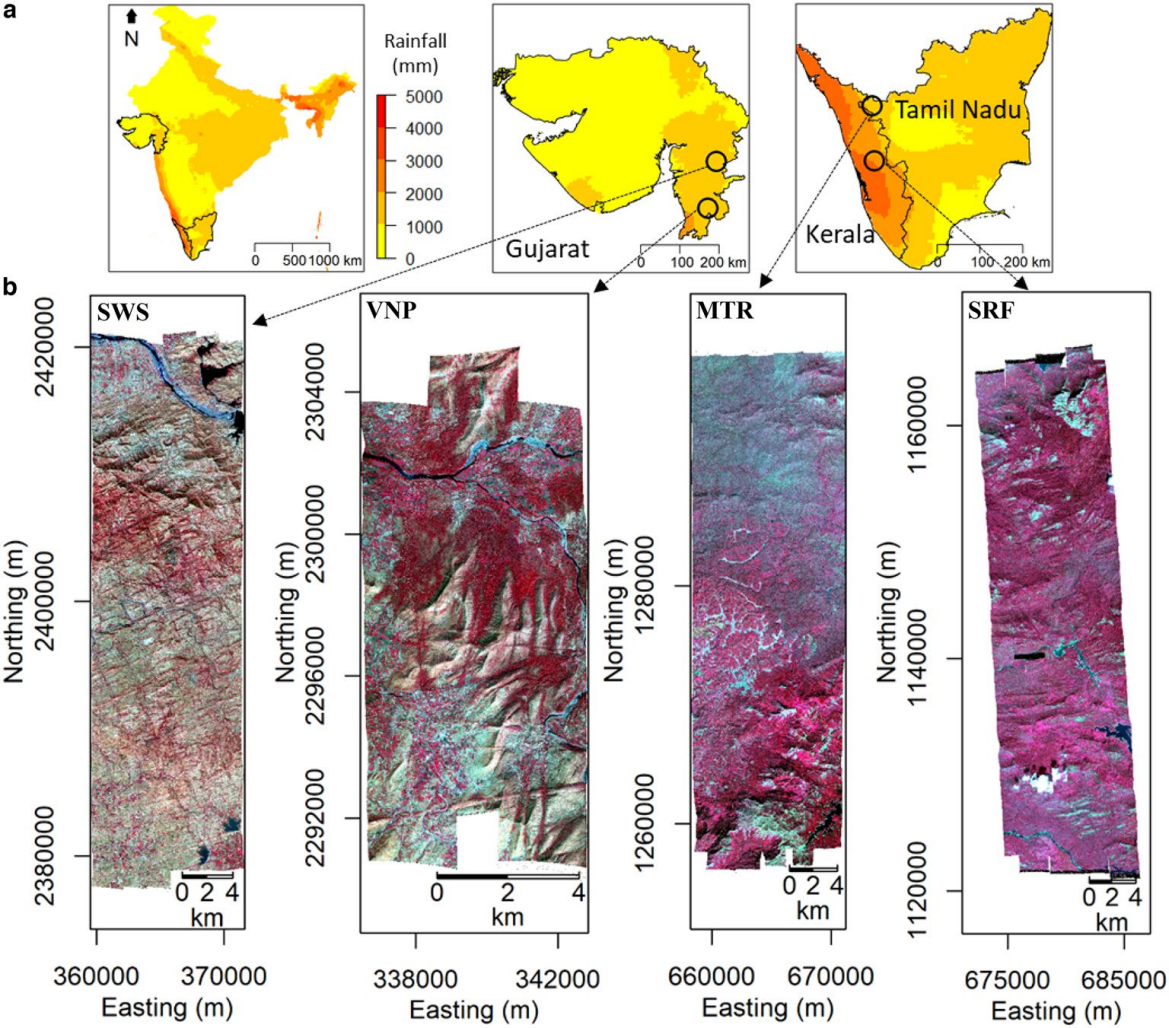
Study area showing the location of protected areas (PAs) along with rainfall gradient in India (**a**). False color composite of each PA (862.28 nm–red; 651.92 nm– green; 551.74 nm–blue) developed from AVIRIS-NG datasets (**b**). Red color represent vegetation, cyan/green represent barren areas and human settlements. The full names of the PAs are SWS (Shoolpaneshwar Wildlife Sanctuary), VNP (Vansda National Park), MTR (Mudumalai Tiger Reserve), and SRF (Sholayar Reserve Forest).

This study has been conducted to address some of these aspects across four PAs namely, Shoolpaneshwar Wildlife Sanctuary (SWS), Vansda National Park (VNP), Mudumalai Tiger Reserve (MTR), and Sholayar Reserve Forest (SRF) (Fig. 1) in India spread over a climate gradient (distinctive gradient observed in the decadal mean of rainfall (1140.13–2757.60 mm) and temperature (24.93–28.08 °C) of the four PAs). Objectives of the study are,

- *Developing accurate abundant tree species maps using AVIRIS-NG datasets*
- *Modeling distribution of these species using SCGLR model across the observed climate gradient*
- *How the observed species heterogeneity impacts functional richness and divergence of their traits*
- *What is the appearance dynamics of an abundant species in dark diversity*

## Results

### Observations from the field study

**Figure 2:**
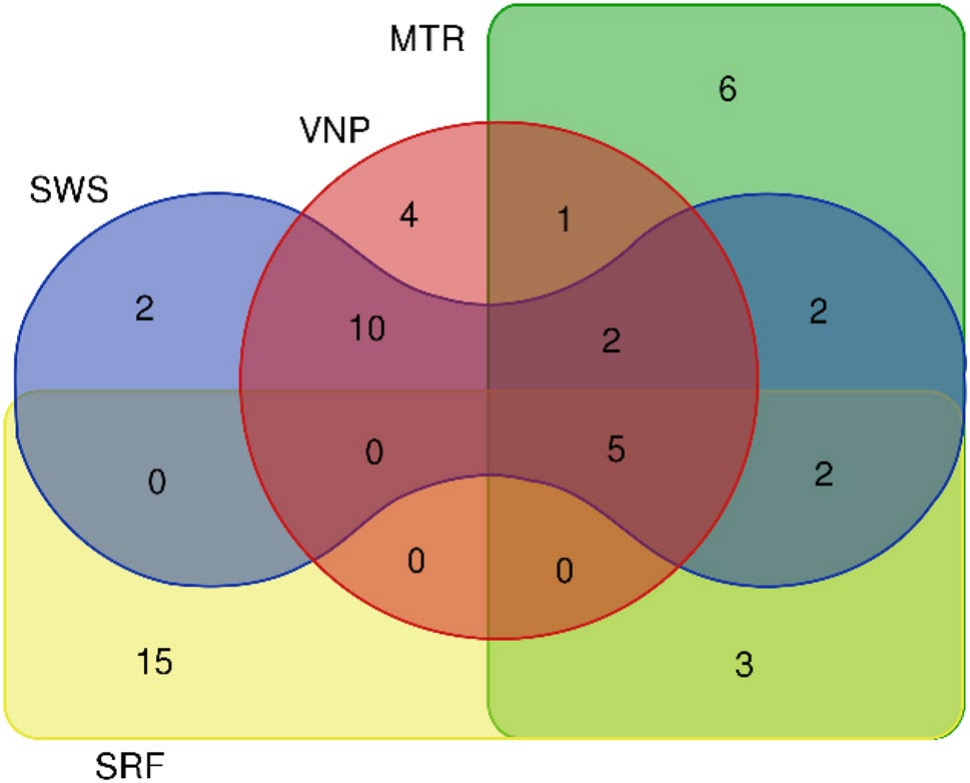
Venn diagram showing the distribution of abundant species as common (at two/three/four PAs) and PA-specific species over four PAs.

A total of 160 tree species belonging to 110 genera and 43 families were recorded over four PAs. The diversity of tree species ranged from 70 to 80 at each PA. The number of abundant species spread (88–93%) across the forest cover of each PA is 24 (SWS), 23 (VNP), 22 (MTR) and 26 (SRF). Amongst the marked abundant species of four PAs, 26 species were common at two PAs at the least and 27 were PA-specific. The number of PA-specific species consistently increased across the gradient and correspondingly common species numbers decreased (Fig. 2). The canopy phenology of tree species showed diverse phases at four PAs. Number of deciduous species decreased and evergreen species increased across the gradient. The number of species belonging to Fabaceae was relatively more and their number decreased across the climate gradient (Supplementary Table S1). The diameter of the trees at 1.37 m height from the ground (diameter at breast height–DBH) ranged between 0.04 and 1.43 m (mean and median values of DBH at each PA, Supplementary Table S2).

### Classification maps of abundant species

**Figure 3:**
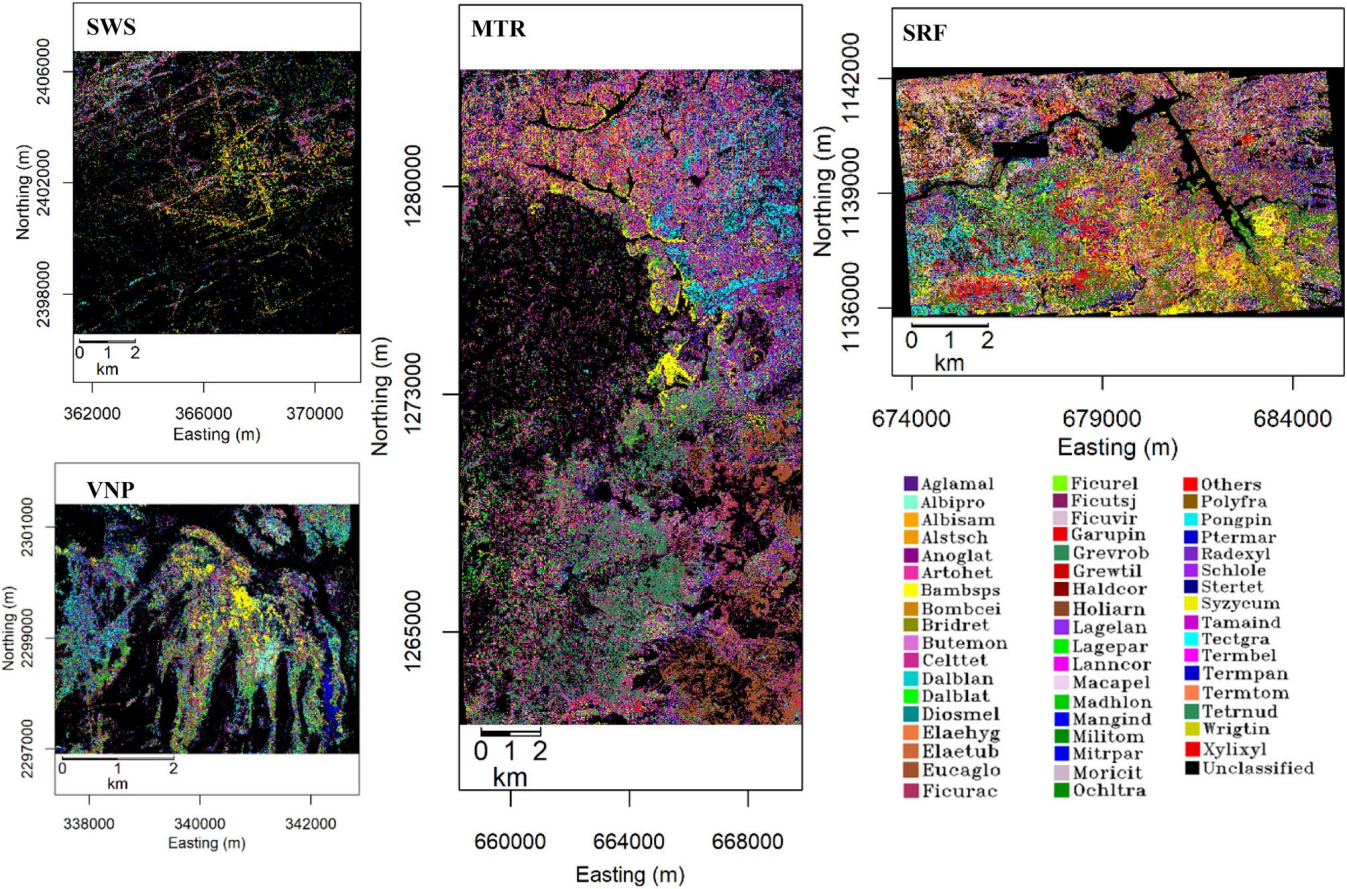
Abundant species classification maps of four PAs using the Random Forest model on the processed subsets of AVIRIS-NG images (Supplementary Table S17 for the complete names of species). Masked regions are shown in black color. RF modelled forest cover area of each PA is 17.23 sq. km (SWS), 10.92 sq. km (VNP), 141.67 sq. km (MTR) and 57.91 sq. km (SRF).

Classification maps of abundant species coming from Random Forest (RF) model run on the subsets of AVIRIS-NG image data of each PA can be seen in Fig. 3. Classification maps of each PA showed a fair overall accuracy ranging from 76.92 to 81.04% with 0.76–0.80 kappa coefficient (Supplementary Table S3). Other accuracy assessment parameters are consistent (Supplementary Table S3, S4–S7 and Extended Data Fig. 1). In agreement with field data, proportion of spread of PA-specific species (Extended Data Fig. 2), and of evergreen species (Extended Data Fig. 3) increased across the climate gradient.

### Floristic composition, climate components, species abundance and assemblage

**Figure 4:**
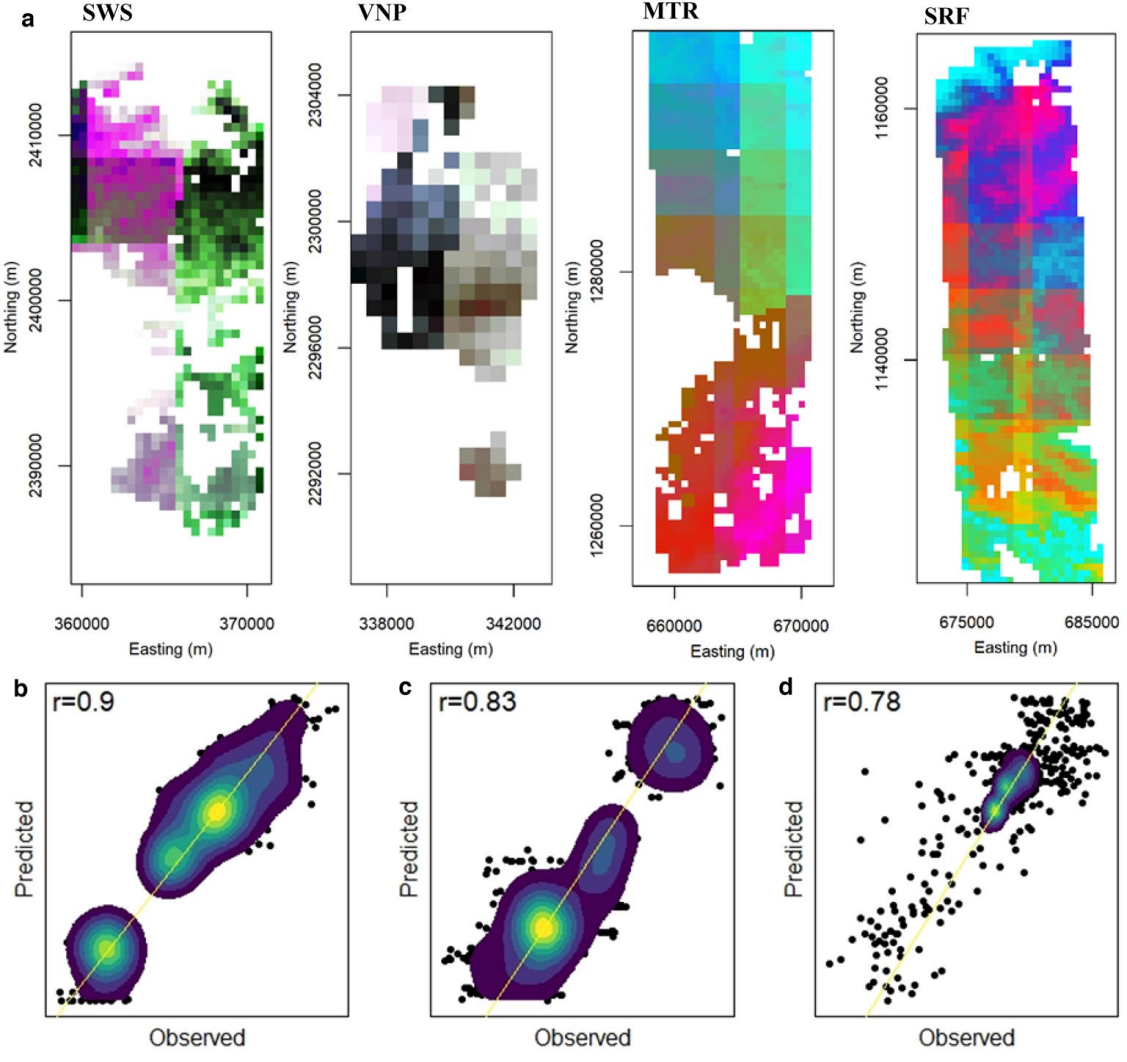
Abundant species composition in four PAs. (**a**)**,** Each PA’s spatial extent of RGB color composite of the first three axes of correspondence analysis (CA) carried out on the projected taxon abundances (n = 53 species; axis 1; red, axis 2; green, and axis 3; blue). (**b**–**d**)**,** Cross-validation outcome between the observed and predicted CA gradients for axis 1 (**b**), axis 2 (**c**) and axis 3 (**d**). The 1:1 line is showed in yellow. Forest cover areas of each PA are 176 sq. km (SWS), 39 sq. km (VNP), 409.5 sq. km (MTR), and 424.25 sq. km (SRF).

The SCGLR model run using species abundance and assemblage data from each 0.005° grid cell of the classification maps of four PAs along with respective cell’s CCs and other abiotic parameters, estimated each species abundance with an overall median Spearman correlation *ρ*_cv_ of 0.71 (ranged from 0.15 to 0.81). A high median value showed that the model picked up differences in the distributional range of a species as well. The first three CCs in SCGLR model accounted for 98.76% of inertia. Critical CCs impacting species assemblages and abundance are mostly related to rainfall (Extended Data Fig. 4). Correspondence analysis (CA) was carried out on the grid cells × observed species abundance matrix to check species assemblages over the noted CA axes. It highlighted important regional floristic gradients (Fig. 4) with high correlation values (*ρ*_cv_ = 0.9, 0.83 and 0.78 for CA axes 1, 2 and 3 respectively). The first and major floristic gradient projected here (CA axis 1) was related to the second predictive CC (Pearson’s r = 0.87). The second projected floristic gradient (CA axis 2) was correlated to CC1 (Pearson’s r = -0.78). The third projected floristic gradient (CA axis 3) showed no particular correlation pattern with climatic components.

### Functional community composition

**Figure 5:**
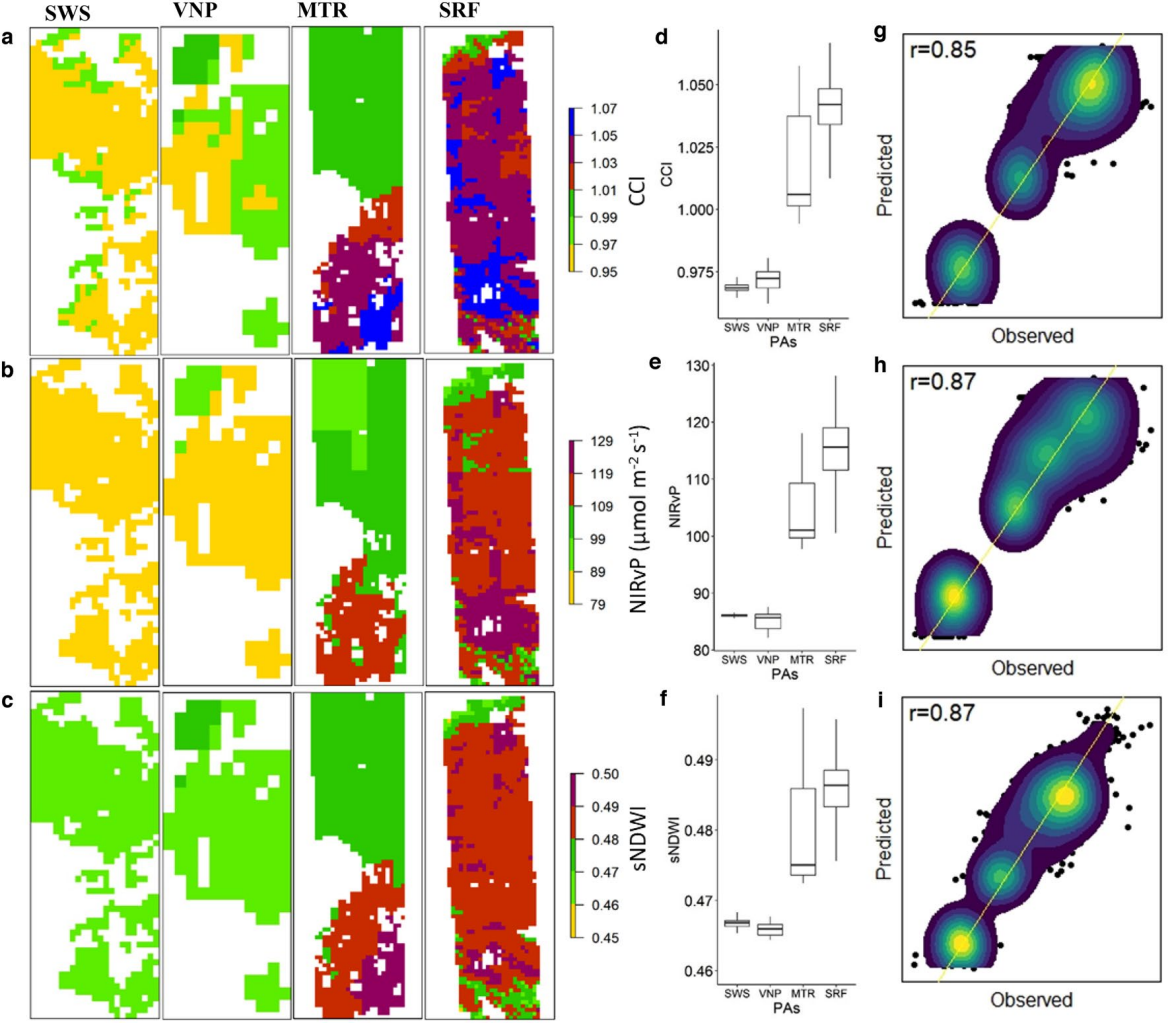
Composition maps of functional traits. Projected community weighted trait values of four PAs (CCI (**a**)**,** NIRvP (**b**) and sNDWI (**c**)) at 0.005° resolution. (**d**–**f**), Box and whisker plots of CWMs of biochemical functional traits. Cross-validation values between the observed and predicted community weighted functional trait values (CCI (**g**), NIRvP (**h**) and sNDWI (**i**)). The 1:1 line is showed in yellow.

CWMs of biophysical (Wood density, Canopy phenology and maximum DBH) and biochemical (CCI, NIRvP and sNDWI) traits were calculated using these forecasted species assemblages and their abundance over classification maps to project the functional composition of abundant tree species over PAs (Fig. 5 and Extended Data Fig. 5). The estimated functional composition of species was orderly for the three biophysical traits (*ρ*_cv_ = 0.52, 0.79 and 0.65) respectively. All the three biochemical traits coming from AVIRIS-NG data outperformed with higher correlation (*ρ*_cv_ = 0.85, 0.87 and 0.87 respectively) between the observed and predicted CWMs of traits.

### Hierarchical cluster analysis, species assemblage types and CWMs

**Figure 6:**
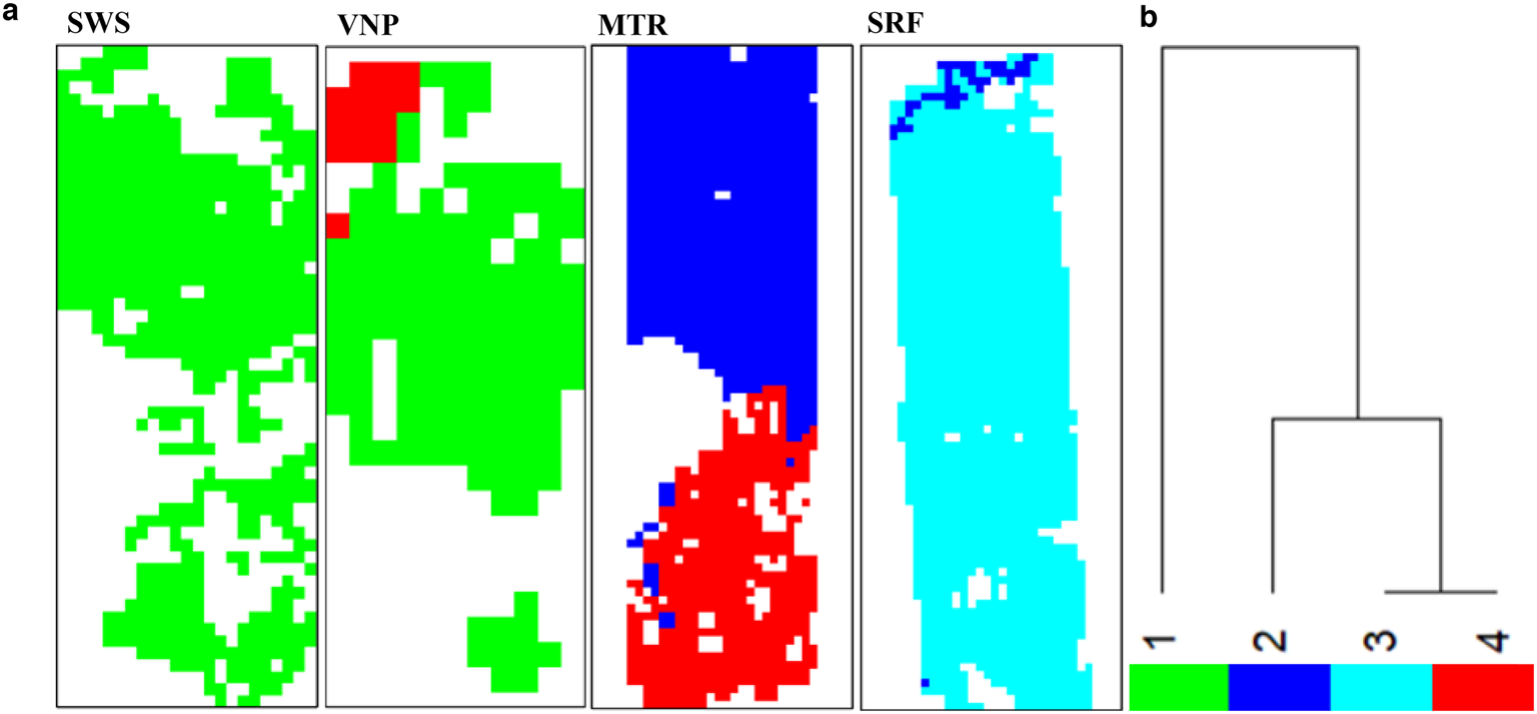
Major assemblage types of abundant species coming from Ward’s hierarchical clustering over PAs (**a**). Cluster dendrogram showing similarity in four species assemblage types (**b**).

The developed SCGLR model on species classification maps was applied to the resampled grid cells of climate variables spread over the entire forest covers of PAs. Hierarchical cluster analysis was performed to estimate floristic variability amongst the observed abundant species over the climate gradient. Euclidean distances between plot scores on the first five CA axes and a Ward’s linkage performed revealed four major species assemblage types (Fig. 6). The number of types chosen was based on the uncertainty values (mean uncertainty per assemblage type ranged between 0 and 0.08%). The number of types of assemblages was minimum at SWS (only one) and the rest of PAs showed two assemblage types. Distinctive demarcation with reference to species assortment and their functional traits was seen between the two assemblage types at these PAs. Species assemblage types of drier and wetter PAs showed dissimilarity with wetter PAs having a greater proportion of PA-specific species. Observed dissimilarity amongst assemblage types is because of the number and spread of evergreen and deciduous species. Similarity in species composition between the same assemblage types at two PAs was different (Type 1 at SWS and VNP with 62% similarity; Type 2 at MTR and SRF with 31% similarity; Type 4 at VNP and MTR with 25% similarity).

Box and whisker plots of CWMs of forest covers of PAs showed consistent increase in the length of the box and of whiskers (Fig. 5d–f) for biochemical traits observed at wetter PAs over the drier ones. Differences in the values between drier and wetter PAs were found significant for the three biochemical traits, and for two of the biophysical traits (DBH and canopy phenology (Extended Data Fig. 6)). Similar consistent feature was not seen in the plots of CWMs of wood density. The median value of trunk DBH was higher in wetter PAs. CWM of canopy phenology showed a positive response over the climate gradient (Extended Data Fig. 5e). CWMs of observed traits of different assemblage types at a PA (noticed at VNP, MTR and SRF) are different indicating the impact of species assortment on trait dynamics (Extended Data Fig. 7 & 8).

### Functional diversity

Functional diversity as measured from functional richness (FRic) and functional divergence (FDiv) for the entire forest cover of each PA showed diverse results. FRic indicating about the volume occupied by the traits values of a community was the lowest at SRF and highest at VNP for the measured traits (Extended Data Table 1). FDiv mentioning about how species abundance gets reflected on the functional trait axis was maximum at SRF for biophysical traits, but for biochemical traits, it is maximum at SWS. Values of the other PAs were variable for the measured traits. Amongst the observed species assemblage types, FDiv values were higher in wetter PAs over the drier ones (Supplementary Table S8).

### Dark diversity

DD calculated based on co-occurrence values of species^1,25^ showed that the number of plots showing DD was much higher at PAs of the wetter region over those in the drier region (Supplementary Table S9 & S10). The mean of abundant species falling in DD per plot across the climate gradient varied from 3 to 5 species (Supplementary Table S11). The appearance of common abundant species in DD was variable (Supplementary Table S12) across PAs. PA-specific species of MTR showed higher representation in DD (Supplementary Table S13) revealing their better co-occurrence ability over the other PA-specific species. Species common to wetter PAs also showed greater presence in DD.

## Discussion

### Abundant species maps

Our study highlights the importance of integration of field studies with remote sensing data for effectively monitoring forest covers in PAs. It attempts to examine the role of species assemblage variability on CWMs of functional traits over a climate gradient. Similar emphasis was laid down earlier^4^ for using remote sensing to extract functional traits data over an environmental gradient. Another report^7^ highlighted the global importance of assessing dominant species, their trait values using field- and remote sensing studies and another emphasized the importance of knowing tree species configuration of forests for their effective management^26^. Observations of these reports reinforce the significance of this study. Using RF model, 62% accuracy for five of the common tree species of a temperate region was reported^26^. A different study^27^ developed a composition map of 23 tree species with an accuracy of 82–87% for two sites in Israel using imaging spectroscopy data with one metre spatial resolution. A recent study^28^ reported classification of tree species of forest covers of United States ecological networks with an individual tree accuracy of 77% for the developed General model. The accuracy of species classification using the RF model in our study is reasonable, given the number of abundant species considered and the spatial resolution of the sensor used. The obtained maps assisted well in extracting large-scale plot data for testing the SCGLR model.

### SCGLR model, distribution dynamics of abundant tree species and CWMs

Similar to previous work^3^, our study provides continuous data on species and functional assemblages of abundant tree species across four PAs along a climate gradient. The tested SCGLR model effectively models and highlights the impact of CCs on the distribution of tropical tree species across these PAs. The composition variability observed among the four assemblage types demonstrates how abundant species are sorted along the climate gradient. From the generated data we can’t say that species shuffling lags behind climate warming^29^. Contrary to previous work^3^, our combination of field and remote sensing data allows us to extract biochemical functional traits, which show greater sensitivity to the observed climate gradient. The importance of observations and inferences coming from this study were emphasized earlier^5^ by mentioning that details of the spatial distribution of species and their traits characteristics are vital for models focusing on climate and landscape disturbance.

Observed dynamics in the CWMs of functional traits revealed the role of intra- and inter-species variability, importance of species heterogeneity in community ecology^30^, and the role of PA-specific species in further filtering of species types^31^. Amongst the five common abundant species over all PAs, SCGLR model projected spread was > 50% for Tectgra and Bambsps (Extended Data Fig. 9) indicating that they can blend easily into different assortments of species over the climate gradient. Our study proves that the retrieval of Essential Biodiversity Variables such as community composition, traits values of species, and their CWMs is possible through the combination of field- and high-resolution remote sensing data^9^ helping in the consistent monitoring of PAs.

### Biochemical traits dynamics

NIRvP measured here exhibits variability among species assemblages across climate gradient. This can be an important proxy to capture spatial patterns of canopy photosynthesis^16^, and a good estimator of gross primary productivity (GPP)^32^ in regions with limited flux tower data availability. As reported^20^, fluctuations in the CCI calculated in this study can effectively record the dynamics of photosynthesis and phenology of deciduous and evergreen species assemblages of the tropics, and can act as a proxy to monitor photosynthetic activity and GPP^33^. The CWMs of NDWI responded positively to the climate gradient and to the proportion of evergreen species in the assemblage. Narrow spread of box and whisker plots and lower median values of NDWI at SWS and VNP revealed how the canopy water content gets impacted at drier PAs owing to senescent vegetation^34^. Box and whisker plots of CWMs coming from the drier PAs showed narrow spread compared to those ones of wetter PAs. This showed that variability in species traits gets reduced at drier regions and trees at wetter PAs maintained relatively higher amplitudes in traits characteristics as observed earlier^35^.

### FRic, FDiv, and DD assessment

The estimated FRic and FDiv showed diverse responses of abundant species to the climate gradient in tune with the observed floristic diversity and assemblage dynamics^36^. FRic indicating the space the three traits own differed with an increase in rainfall from SWS to SRF. Varied values of FRic seen at four PAs could be because of the divergence in the functional traits of species^37^. SRF showed higher FDiv over the other three PAs showing that the spread of species abundance across the functional traits axes was greater. FRic and FDiv measurements provide insights into how tree species are distributed in a multidimensional functional space relevant to the local climate. Unlike the reports coming from a grassland study^38^ our work empirically demonstrates that functionally distinct tree species can effectively coexist and sort themselves across the climate gradient.

DD indicates the rehabilitation ability^1,39^ of PAs across the climate gradient. Common abundant species over the four PAs were seen falling in DD over > 15% of the plots sampled showing their accommodative nature. Variability in the appearance of abundant species in DD across PAs indicates their sensitive approach to climate gradient. Inputs coming from this study can assist in steering the management of PAs^1^ through the selection of tree species that can easily establish because of their ease of co-occurrence with other species.

### Implications of this study

This study reveals the potential of combining field- and remote sensing data to better understand how forest tree cover in PAs responds to the climate gradient. CWMs of functional traits, FRic and FDiv provide valuable insights into the response of tropical tree species to their surroundings. Our study addresses important aspects of macro ecology^40^, including floristic intricacies and trait data from remote sensing^41^, contributes to the understanding of ecological processes by providing inputs for modelling and projecting future scenarios^42^ at the spatial scales of PAs whose vulnerability to climate change can have significant ecological consequences. Inputs from DD can be more affective in the reforestation of PAs. Regular and periodic availability of remote sensing data from Hyperspectral InfraRed Imager (HyspIRI)^8,43^, Earth surface Mineral dust source InvesTigation (EMIT)^44^ and Global Ecosystem Dynamics Investigation (GEDI)^45^ can generate precise inputs over different spatial scales, enhancing the development of ecological models for a deeper understanding of forest cover functionality under various climate dynamics.

## Methods

### Study area

The study was conducted in four protected areas (PAs) of India, namely, Shoolpaneshwar Wildlife Sanctuary (SWS), Vansda National Park (VNP), Mudumalai Tiger Reserve (MTR), and Sholayar Reserve Forest (SRF) (Fig. 1). All the four PAs are with variable human activities which was observed to be more in SWS and lesser at SRF in relative terms. SWS and VNP fall in the western India and MTR and SRF fall in the southern India (Fig. 1) Owing to the lat/long variation, climatic conditions are different. PAs at western India relatively receive lesser rainfall while MTR and SRF receive higher rainfall. This variability in rainfall coupled with temperature dynamics influenced tree cover distribution. The climate components (CCs) and topographic conditions of the four PAs (drivers of floristic gradient) are given in Supplementary Table S14. Vegetation type varied from tropical dry deciduous to tropical evergreen forest over the four PAs^46^. Drier PAs (SWS and VNP) consisted mostly of deciduous species with few evergreen ones, whereas wetter PAs (MTR and SRF) supported deciduous ones with a higher proportion of evergreen species. Schematic representation of the methodology has been shown in Supplementary Fig. 1.

### Climate and topographic data

We used Climate Hazards group Infrared Precipitation with Stations (CHIRPS) data for rainfall^47^ and European ReAnalysis-Interim (ERA-Interim) data for temperature^48^. Spatial resolution of rainfall data was 0.05° (5.12 km) and for temperature it was 0.125° (12.5 km).The spatial resolution of climate (CHIRPS and ERA-Interim) and elevation data were resampled to 0.005° grid cells using Nearest Neighbor method. For this study, we considered six CCs of rainfall and temperature over a period of 10 years (Supplementary Table S15). Observed climate data of rainfall and temperature showed marked gradient across the four PAs, SWS and VNP falling in a drier region and MTR and SRF falling in a wetter region relatively with higher rainfall. Topographic/elevation data were obtained from Cartosat 1 with a spatial resolution of 30 m (National Remote Sensing Centre, https://bhuvan-app3.nrsc.gov.in/data/download). Soil types were obtained from Harmonized World Soil Database (HWSD)^49^ and categorized the four PAs into two classes: Nitisols and others. We assigned the values of six CCs, elevation and soil type to each of the 0.005° grid cells. As reported in several studies these variables shape the species distributions and impact community structure^50,51^.

### Field data sampling

Field visits were conducted at three PAs (SWS, VNP, and MTR) concurrent to the dates of AVIRIS-NG flight visits in 2016 (within a 7-day timeframe). Due to the restrictions in accessibility, the same was not possible at SRF. It was done later. Tree species sampling protocol followed at four PAs was the same. Both quantitative and qualitative parameters of forest tree covers were recorded as described in earlier publications^52,53^. Quadrats of 8 m × 8 m dimensions were placed randomly across the accessible trails of forest covers, and tree species found in each quadrat were identified at the species level with the help of local forest department personnel, flora, and published records. Tree abundance and their biophysical parameters were measured. Height was measured using a Vertex hypsometer (Haglof, Vertex IV). Diameter of trees (at 1.37 m above ground level) and canopy spread were measured using a measuring tape. Tree species with > 4 cm diameter at breast height (DBH) were recorded in the study. We also marked the Global Positioning System (GPS) location of tree species having canopy spread > 4 m. The recorded tree species were divided into different categories such as evergreen and deciduous species, common (seen at more than one PA), and PA-specific (seen only at one PA).

### AVIRIS-NG data acquisition and processing

Airborne Visible/InfraRed Imaging Spectrometer Next Generation (AVIRIS-NG) is a new generation airborne sensor of National Aeronautics and Space Administration (NASA) developed by Jet propulsion laboratory (https://aviris-ng.jpl.nasa.gov/). A flight carrying the AVIRIS-NG sensor was flown over the four PAs in India between January 2016 and February 2016 as a part of a joint campaign between the Indian Space Research Organization (ISRO) and NASA^54^. Details of the AVIRIS-NG image data acquisition are given in Supplementary Table S16. The advanced features of this sensor include high spatial (4 m) and spectral resolution (5 nm) covering solar radiation ranging from 380 to 2510 nm wavelengths. The total number of flight lines and geographical area covered at each of the PA were six and 501.88 sq. km (SWS), three and 82.36 sq. km (VNP), 10 and 540.70 sq. km (MTR), and eight and 493.70 sq. km (SRF). Sensor calibration and atmospheric correction^55–57^ were performed at the Phil Townsend lab, University of Wisconsin, Madison, USA (code available at https://github.com/EnSpec/HyTools-sandbox). The topographically and Bidirectional Reflectance Distribution Function (BRDF) corrected lines of each PA were mosaicked into a single image in the Environment for Visualizing Images (ENVI v 5.3) software following the standard protocol. A subset for each PA was extracted as region of interest from the respective mosaicked image for tree species classification. For analyses purpose, 366 usable bands were retained from the available 425 bands by removing noisy bands (< 411 nm) and water absorptions bands (1348–428, 1778–1949 nm). Normalized Difference Vegetation Index (NDVI) was used to mask non-forest areas with threshold values of 0.4 (SWS and VNP), and 0.6 (MTR and SRF), (Reasoning for different NDVI values, other information can be seen in ref.^52,53^).

### Abundant species classification maps using random forest (RF) model

Generated field data showed that 21–25 species were seen repeatedly at each PA covering > 80% of the forest cover (Supplementary Table S1). These are considered as abundant species. Other than these, four to six species at each PA collectively were seen to spread over 8–10% of forest cover. The number of individuals of each of these species recorded in the field surveys was relatively less. Variability in the physiognomy and canopy spectra of these species appeared lesser. These were combined and designated as a class, ‘others’. The abundant species along with ‘others’ occupied 88–93% of forest cover in each PA. For commonality in expression the class ‘others’ was referred as an abundant species. The number of abundant species considered for each PA was 24 (SWS), 23 (VNP), 22 (MTR) and 26 (SRF). Shape file of crown-level reflectance spectra of these abundant tree species were extracted for the classification from each PA using GPS location of individual trees of species from field records. Pure patches of species (more than two individuals) were seen and their GPS positions were considered. The mean spectra of each species class were plotted together for each PA. After visual observation of spectral region discrimination of each species, 226 bands from four different spectral regions were selected (550–650 nm, 750–1250 nm, 1500–1750 nm and 2000–2250 nm)^58^. Brightness normalization (BNORM, a reflectance normalization method) was used for brightness gradient correction of 226 spectral bands^59^. We further applied Minimum Noise Fraction (MNF) transformation^60^ to reduce the spectral data dimensionality and to obtain uncorrelated components from 226 brightness normalized spectral bands. A simple random sampling technique with replacement was used to divide the extracted canopy spectra into 75% training and 25% testing samples. We applied Synthetic Minority Oversampling TEchnique (SMOTE) to the training samples^61^ to overcome the unbalanced classification problem. After this step a supervised RF classification^62^ was carried out using the MNF-transformed spectra of training samples of each species with caret^63^, raster^64^ and themis^65^ packages in R ver. 4.0.5^66^. The RF model was tuned using different values of tuning parameters (mtry and ntree) at each PA. The number of MNF bands used, their eigenvalues and RF tuning parameter values are given in the Supplementary Table S3 for the four PAs. The resulting abundant species classification maps went through an accuracy assessment procedure in which overall accuracy, kappa coefficient, out-of-bag (OOB) error rate, producer’s and user’s accuracies were calculated for all the species classes.

### SCGLR model, hierarchical clustering of grid cells

We overlaid 0.005° (0.5 × 0.5 km) grid cells on the developed abundant species map of each PA to generate plot data. From each 0.005° grid cell, three 0.5 ha plot data were extracted from the four PAs. Each plot extracted has at the least 70% pixels occupancy with forest tree cover. The minimum number of plots depends on the spatial resolution of gridded data^67^. Here we kept three plots in each grid cell because of the high spatial resolution and to ensure that all grid cells have uniform sampling. The occupancy of grid cells with trees is relatively sparse at SWS compared to the other three PAs. The extracted data of classified pixels coming from each plot were extrapolated to species abundance data based on the canopy spread values of each species recorded during the field study. Following the methodology given in ref.^3^, the abundance data of these tree species in three plots were aggregated in each of the 0.005° grid cells across the abundant species maps developed. The reference dataset now contains species distribution values of 53 abundant species across four PAs (one is of ‘others’ at each PA, Supplementary Table S17) for 1006 of 0.005° grid cells (~ 251.5 sq. km of area).

The SCGLR model^3^ was tested using this dataset. SCGLR model integrates Partial least squares (PLS) regression with generalized linear model estimation in the multivariate context. SCGLR model can be applied on both simulated and real data^68^. The model produces the best latent proportions towards modeling a group of responses from a group of explanatory variables. Over-fitting is avoided^69^. Species abundance data coupled with each grid cell’s six CCs, elevation and soil type data were given as inputs to the SCGLR model to project the distribution of abundant species across the RF model developed maps. Ward’s hierarchical clustering was performed^70^ on coordinates of grid cells which divided the dataset into 23 spatial clusters (Extended Data Fig. 10). Leave-one-block-out cross-validation was carried out on these 23 spatial clusters to evaluate the predictive power of the SCGLR model. The model was validated using nonparametric Spearman’s rank correlation between observed and predicted abundance data. Observed and predicted individual species abundance correlation were calculated. Correspondence analysis (CA) was carried out on grid cells × observed species abundance matrix to check species assemblages over the noted CA axes. Values on each CA axis were collected by estimating the grid cell × predicted abundance matrix of species in the noted CA planes. This has enabled us to evaluate the performance of the model to project floristic gradients across the four PAs.

### Generation of functional traits data and CWMs

The functional traits considered in this study were biophysical (Wood density, Canopy phenology and maximum DBH) and biochemical (CCI, NIRvP and sNDWI) attributes. The wood density of each species was obtained from available databases^71,72^. The canopy phenology of each species was demarcated into evergreen and deciduous (Supplementary Table S17). This was obtained from field observations and subsequently corroborated with published records. The DBH of the trunk was measured during field study. Following ref.^73^, maximum DBH of a species at each PA was estimated as a mean of the two largest individuals. CCI, NIRvP and sNDWI were extracted from spectral reflectance data (Supplementary Table S18)^15–17,21^. Wood density and canopy phenology values were the same for abundant species located at more than one PA. The other four parameters for all the species were PA-specific. Multiple values of CCI, NIRvP and sNDWI of each species at each PA were arrayed, and 95% confidence interval values were retained for further analysis. The mean of each parameter (CCI, NIRvP and sNDWI) for each species located at each PA were independently calculated. Mean values of a species located in more than one PA were summed up, and its mean was calculated. Mean values of PA-specific species were considered as it is. These mean values of functional traits of each species were used for estimating CWMs and for developing functional maps. Correlations were also obtained between CWMs of observed and predicted abundant species assemblages of four PAs.

### Extrapolation of the developed model across the forest cover of PAs, CWMs of traits, species assemblage types

Resampled 0.005° grid cells of climate and elevation data were aligned and cropped to the physical extent of PAs as covered by AVIRIS-NG sensor flight. Non-forest cover of each of the 0.005° grid cell was masked. The final product resulted in 4195 of 0.005° grid cells of forest, (~ 1048.75 sq. km of area). The developed SCGLR model was applied on these 4195 grid cells to obtain the spatial spread of abundant species, and CWMs of functional traits across the forest covers of PAs. CA was performed between grid cells × predicted abundance of species matrix. Ward’s hierarchical clustering on the first five CA axes resulted in four species assemblage types. The number of assemblage types was determined based on uncertainty values. Box and whisker plots were developed for the CWMs of each trait of each PA, and for each of the species assemblage type.

### Functional diversity metrics

Two functional diversity metrics, functional richness (FRic) and functional divergence (FDiv) were calculated using the “FD” package^74^ in R ver. 4.0.5. FRic represents the convex hull volume of three functional traits (three of biophysical and another three of biochemical)^75^. FDiv represents the spread of species abundance along the axis of functional trait with the community^76^. Abundant species assemblages in each grid cell across each of the PA (704 for SWS, 156 for VNP, 1638 for MTR and 1697 for SRF, totaling 4195 grid cells) were utilized to calculate FRic and FDiv. Both the functional diversity indices were also calculated for the grid cells representing the four species assemblage types. Mean values of FRic and FDiv were calculated for each PA and for each species assemblage type.

### Measuring dark diversity (DD)

We used presence/absence matrix of species coming from 0.5 ha plots extracted from the RF model developed subsets of four PAs to estimate DD using the Beals’ co-occurrence index^25^ which is the most widely used method^2,24^. Abundant species diversity in each of the 0.5 ha plots considered for this analysis is greater than five^39^. DD was calculated using the “DarkDiv” package^77^ in R ver. 4.0.5^66^. Co-occurrence probability of species in all plots was calculated (following the published code^78^) and 5% quantile probability threshold was used to determine whether a species belongs to DD or not^1^. Number of species falling in DD were computed for drier and wetter PAs and also summed up for all PAs. A list of species falling in DD of > 15% of the plots extracted was made to see variability, if any, amongst abundant species role as a part of DD.

### Supplementary Information

Supplementary Information.

## Data Availability

The datasets used and/or analyzed during the current study available from the corresponding author on reasonable request.
