# Supplementary material for: Integrating field- and remote sensing data to perceive species heterogeneity across a climate gradient
Source: Sci Rep. 2024 Jan 2;14:42. doi: 10.1038/s41598-023-50812-y (PMC10761838; doi:10.1038/s41598-023-50812-y)
Supplement: Supplementary file 1 — Supplementary Information. [file 41598_2023_50812_MOESM1_ESM.docx]

**Extended Data and Supplementary Information**

**Integrating field- and remote sensing data to perceive species heterogeneity across a climate gradient**

Amrita N. Chaurasia, Reshma M. Parmar, Maulik G. Dave and N. S. R. Krishnayya*

Ecology Laboratory, Department of Botany, The Maharaja Sayajirao University of Baroda, Vadodara 390002, Gujarat, India.

**Extended Data**


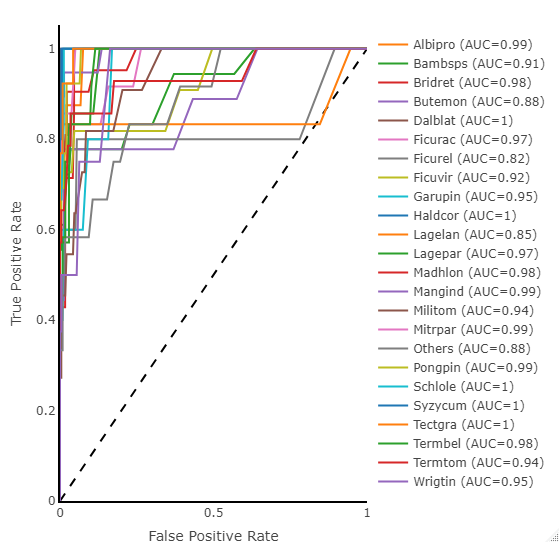

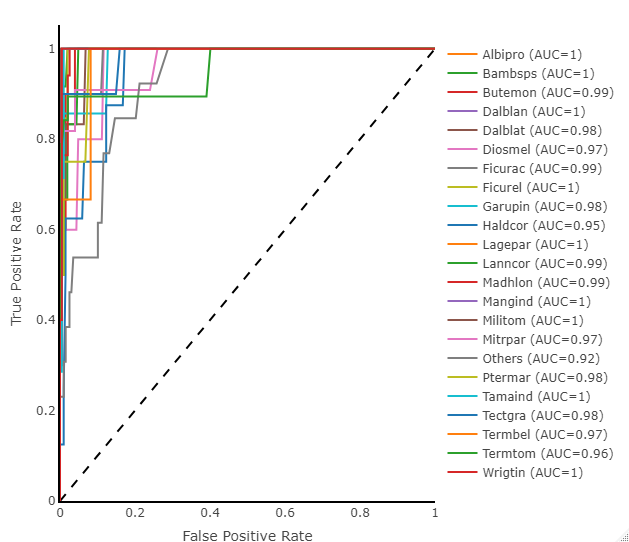

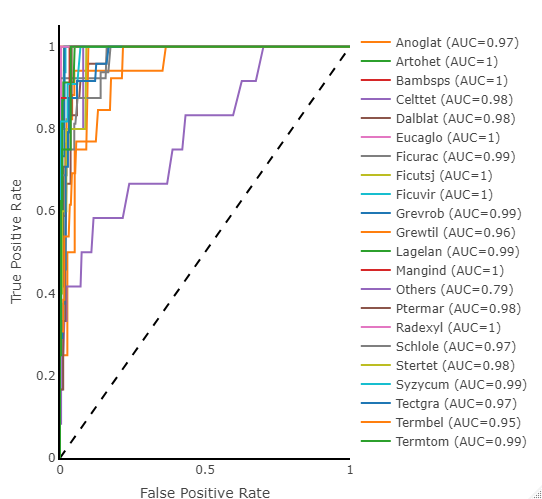

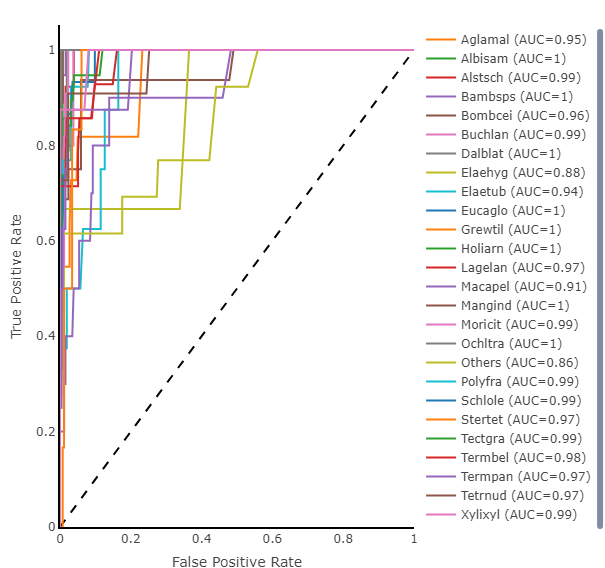


**SWS**

**VNP**

**MTR**

**SRF**

**Extended Data Fig. 1|Receiver operating characteristic (ROC) curve of each species considered for RF classification and their derived area under the curve (AUC) of four PAs.** The mean AUC value for each PA was 0.95 (SWS), 0.98 (VNP), 0.98 (MTR), and 0.97 (SRF).


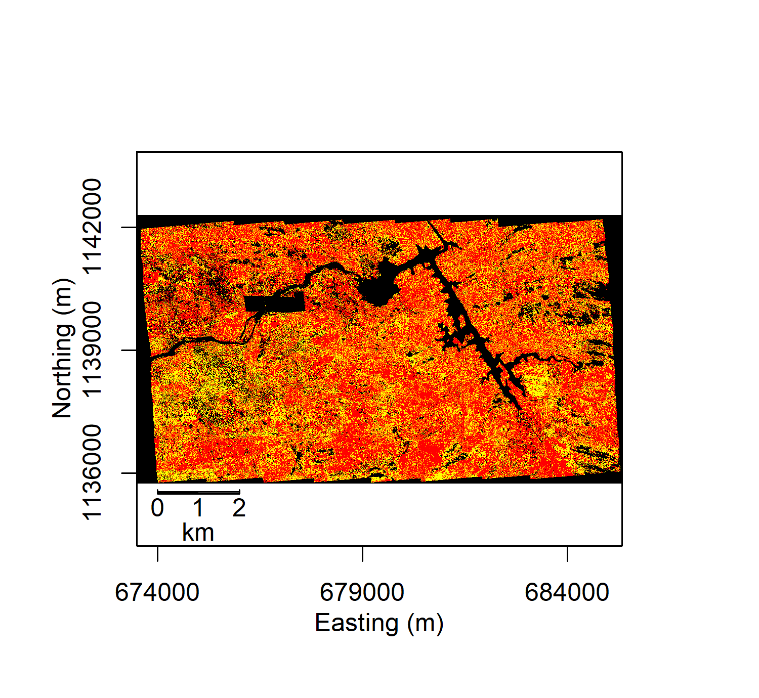

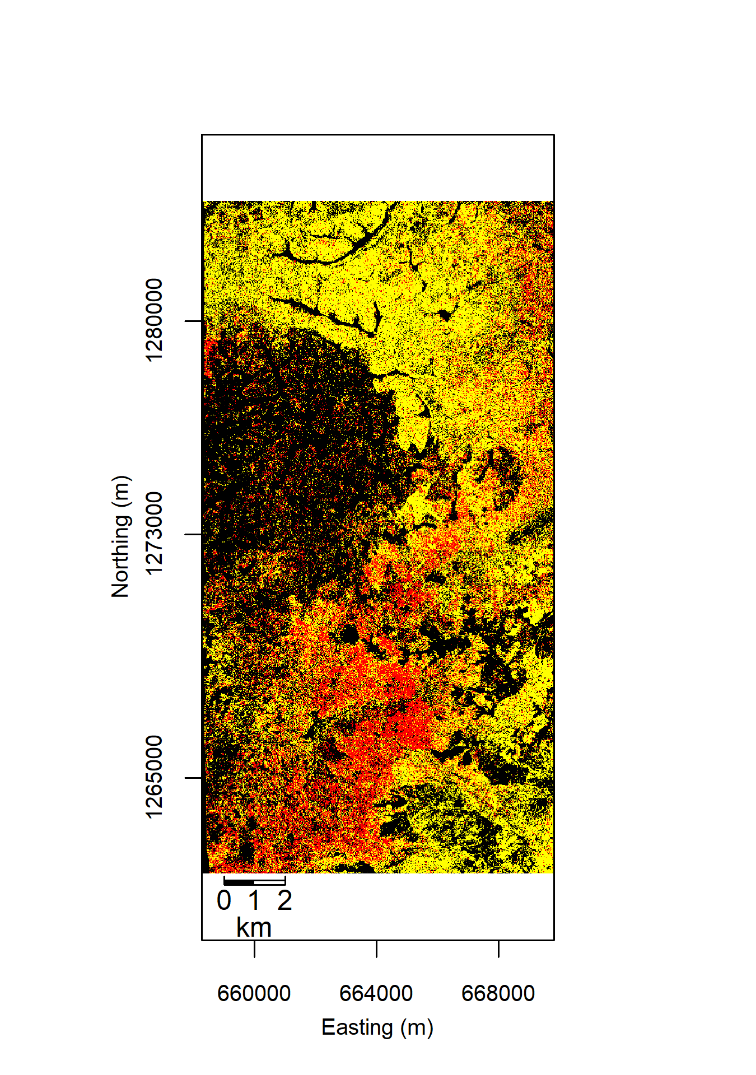

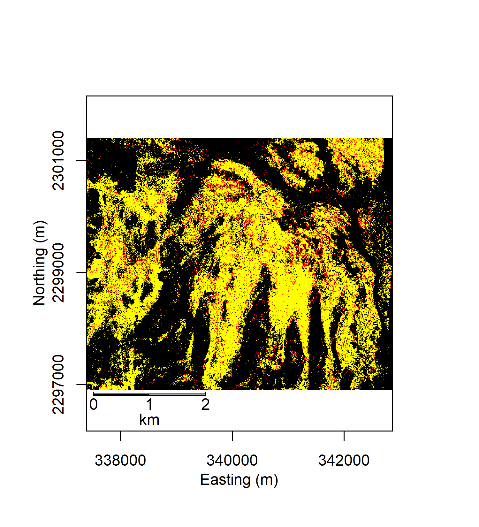

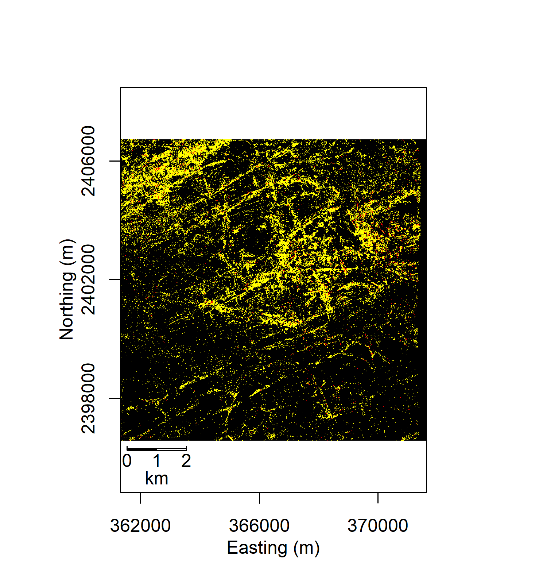


**SWS**

**VNP**

**MTR**

**SRF**


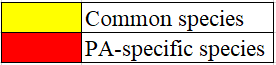


**Extended Data Fig. 2| Progressive spread of PA-specific species across the climate gradient.**


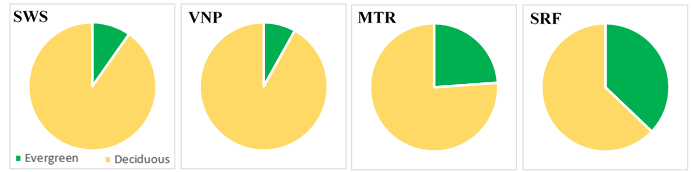


**Extended Data Fig. 3|The proportion of distribution of evergreen species across the observed climate gradient.**


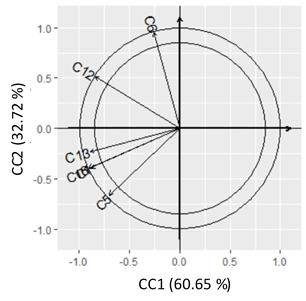

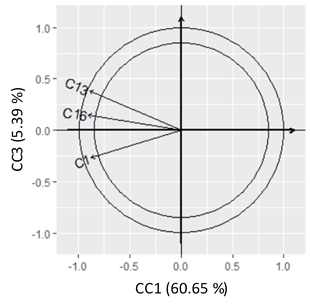


**a**

**b**

**Extended Data Fig. 4| Correlation plots between climate components of SCGLR model. (a) planes 1**–**2 and (b) planes 1**–**3. Expansion of short forms, supplementary Table S15.**


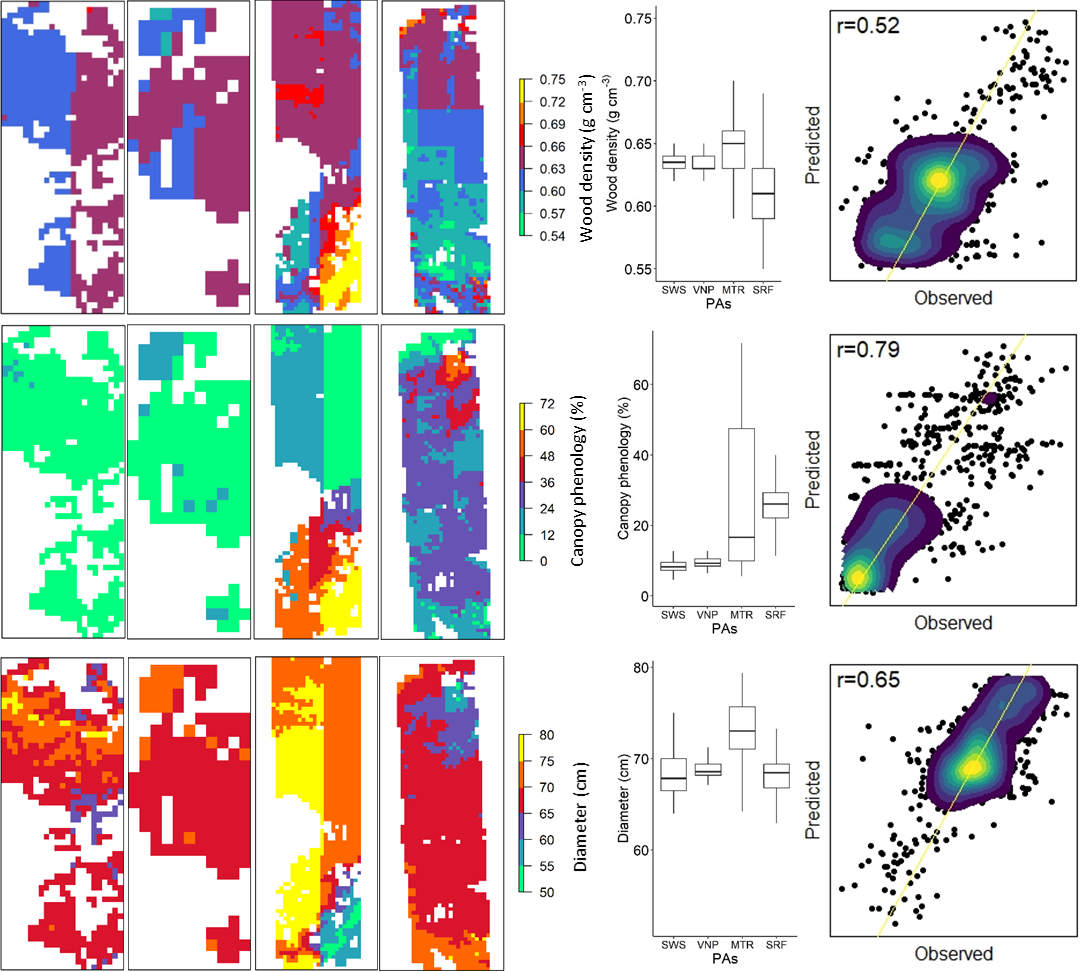


**SWS**

**VNP**

**MTR**

**SRF**

**a**

**b**

**d**

**e**

**g**

**h**

**f**

**c**

**i**

**Extended Data Fig. 5| Composition maps of functional traits.** Projected community weighted trait values of four PAs (Wood density **(a)**, Canopy phenology **(b)**, and Diameter **(c)**) at 0.005^⁰^ resolution. **d**–**f**, Box and whisker plots of CWMs of biophysical functional traits. Cross-validation values between the observed and predicted community weighted functional trait values (Wood density **(g)**, Canopy phenology **(h)**, Diameter **(i)**). The 1:1 line is showed in yellow.


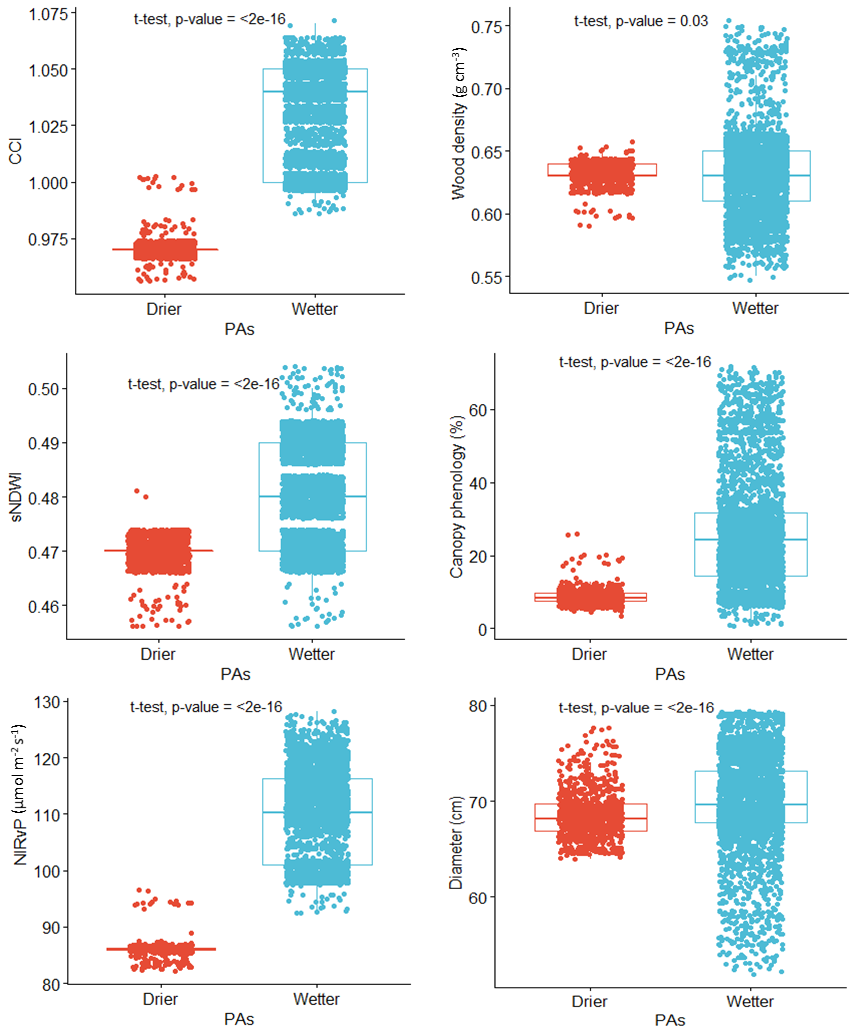


**a**

**d**

**b**

**e**

**c**

**f**

**Extended Data Fig. 6|** **Significance test (t-test) performed between functional traits values of drier (n = 860) and wetter (n = 3292) PAs.** Biochemical functional traits **(a**–**c)** and Biophysical functional traits **(d**–**f)**.


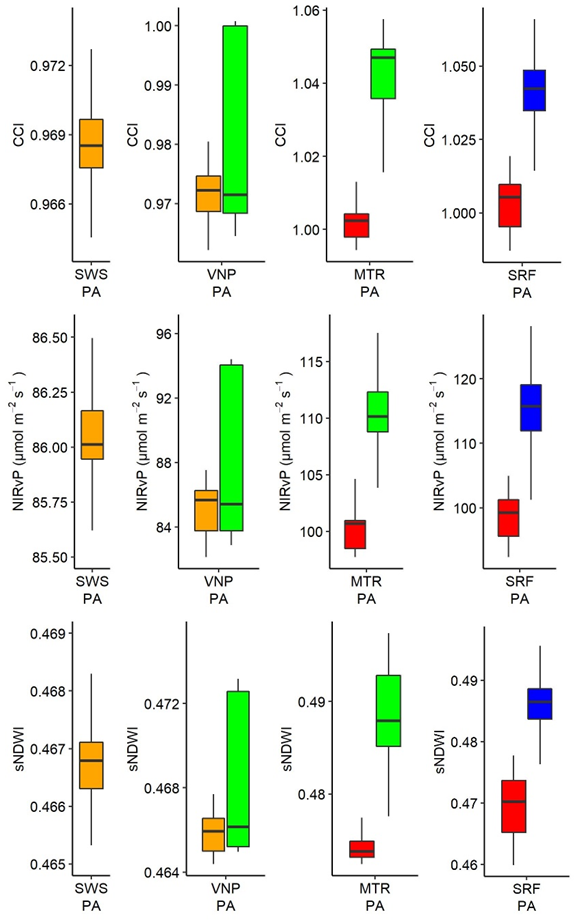

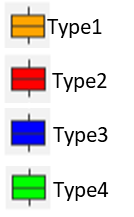


**Extended Data Fig. 7|** **Variation in community weighted mean of biochemical parameters in four species assemblage types of four PAs.** The box and whiskers plot represents the median, the 25^th^ and 75^th^ percentiles, and the minimum and maximum values in the data.


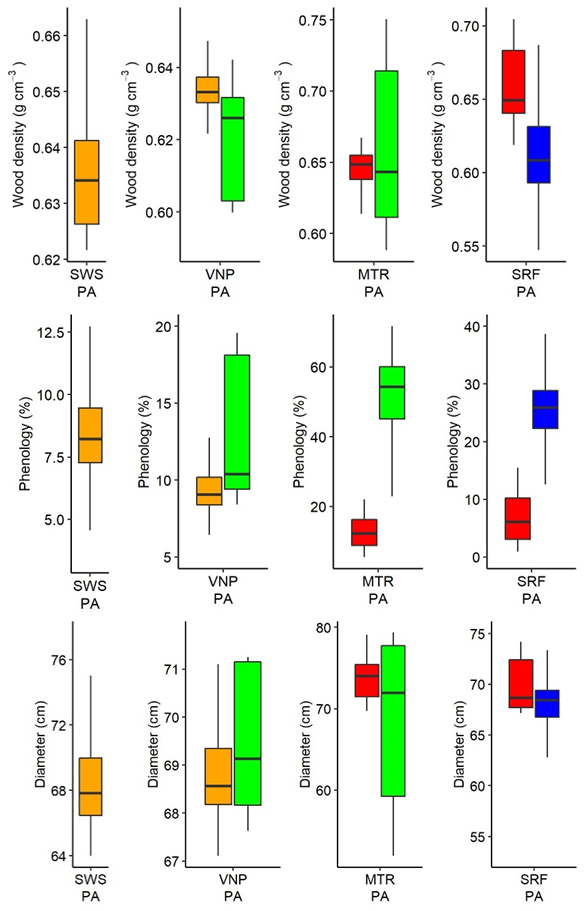

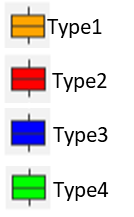


**Extended Data Fig. 8|** **Variation in community weighted mean of biophysical parameters in four species assemblage types of four PAs.** The box and whiskers plot represents the median, the 25^th^ and 75^th^ percentiles, and the minimum and maximum values in the data.

| **Extended Data Table 1\| Mean values of functional diversity (FRic and FDiv) of the measured biophysical and biochemical parameters of the entire forest covers.** | | | | |
| --- | --- | --- | --- | --- |
| **PAs** | **Biophysical** | | **Biochemical** | |
|  | **FRic** | **FDiv** | **FRic** | **FDiv** |
| SWS | 15.31 | 0.74 | 6.23 | 0.78 |
| VNP | 19.89 | 0.67 | 7.20 | 0.76 |
| MTR | 15.43 | 0.77 | 5.78 | 0.72 |
| SRF | 11.83 | 0.81 | 5.82 | 0.76 |

**Extended Data Fig. 9| Proportional spread of five common abundant species coming from extrapolation of SCGLR model over forest covers of PAs (Supplementary Table S17 for the complete names of species).**


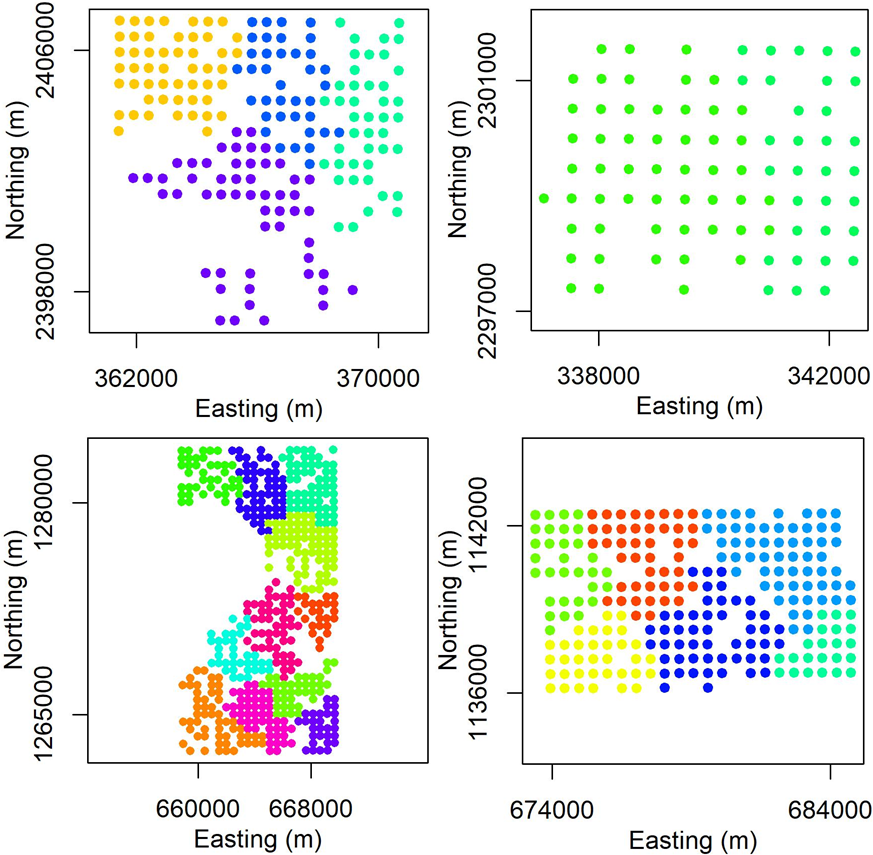


**SWS**

**VNP**

**MTR**

**SRF**

**Extended Data Fig. 10| 23 spatial clusters identified through Ward’s hierarchical clustering method performed on grid coordinates.** These clusters were used for leave-one block out cross-validation. All but one of the clusters were used to train a model, which was then applied to predict data excluded from model calibration.

| Table S1\| Grouping of the observed tree species across four Protected areas (PAs). | | | | | |
| --- | --- | --- | --- | --- | --- |
| All species found in field survey | | | | | |
|  | **SWS** | **VNP** | **MTR** | **SRF** | **Total of four PAs** |
| Total Species | 73 | 70 | 80 | 73 | 160 |
| Number of Families | 31 | 29 | 30 | 29 | 43 |
| Number of species in Fabaceae | 20 | 19 | 14 | 12 | 27 |
| Abundant species | | | | | |
| Total abundant species | 23 | 22 | 21 | 25 | 52 |
| Deciduous species | 19 | 20 | 16 | 14 | 36 |
| Evergreen species | 4 | 2 | 5 | 11 | 16 |
| Common species | 21 | 18 | 15 | 10 | 25 |
| PA-specific species | 2 | 4 | 6 | 15 | 27 |
| % occupancy abundant species | 85.27 | 84.59 | 80.21 | 85.45 |  |
| % occupancy abundant species along with others | 92.11 | 93.02 | 86.85 | 90.79 |  |

**Supplementary Information**

| Table S2\| Mean and median of trunk DBH of trees of abundant species at four PAs. | | |
| --- | --- | --- |
| PAs | **Mean (m)** | **Median (m)** |
| SWS | 0.39 | 0.34 |
| VNP | 0.36 | 0.34 |
| MTR | 0.61 | 0.60 |
| SRF | 0.48 | 0.46 |

| Table S3\| Accuracy values and classification parameters of the Random Forest model applied over four PAs. | | | | |
| --- | --- | --- | --- | --- |
| Parameters | **SWS** | **VNP** | **MTR** | **SRF** |
| Overall accuracy (%) | 76.92 | 81.04 | 80 | 77.32 |
| Kappa coefficient | 0.76 | 0.80 | 0.79 | 0.76 |
| Producer's accuracy (%) | 76.78 | 79.24 | 75.75 | 76.9 |
| User's accuracy (%) | 76.97 | 80.18 | 79.5 | 76.92 |
| OOB error (%) | 11.13 | 9.27 | 11.23 | 14.08 |
| MNF bands | 20 | 15 | 20 | 20 |
| mtry | 2 | 2 | 2 | 2 |
| ntree | 300 | 500 | 700 | 800 |

| Table S4\| Error matrices for random forest classification of SWS. The top row of classes represents the reference trees, and the left column represents classified trees. | | | | | | | | | | | | | | | | | | | | | | | | | | |
| --- | --- | --- | --- | --- | --- | --- | --- | --- | --- | --- | --- | --- | --- | --- | --- | --- | --- | --- | --- | --- | --- | --- | --- | --- | --- | --- |
| Prediction | Albipro | Bambsps | Bridret | Butemon | Dalblat | Ficurac | Ficurel | Ficuvir | Garupin | Haldcor | Lagelan | Lagepar | Madhlon | Mangind | Militom | Mitrpar | Others | Pongpin | Schlole | Syzycum | Tectgra | Termbel | Termtom | Wrigtin | Total | User's accuracy (%) |
| Albipro | 7 | 0 | 0 | 0 | 0 | 0 | 0 | 0 | 0 | 0 | 0 | 0 | 0 | 0 | 0 | 0 | 0 | 1 | 0 | 0 | 0 | 0 | 0 | 0 | 8 | 87.50 |
| Bambsps | 0 | 12 | 0 | 0 | 0 | 0 | 0 | 0 | 0 | 0 | 1 | 0 | 0 | 0 | 1 | 0 | 0 | 0 | 0 | 0 | 0 | 0 | 0 | 1 | 15 | 80.00 |
| Bridret | 0 | 2 | 7 | 1 | 0 | 0 | 0 | 0 | 0 | 0 | 0 | 0 | 0 | 0 | 0 | 0 | 2 | 0 | 0 | 0 | 1 | 0 | 0 | 2 | 15 | 46.67 |
| Butemon | 0 | 1 | 0 | 7 | 0 | 0 | 0 | 0 | 0 | 0 | 0 | 0 | 0 | 0 | 1 | 0 | 0 | 0 | 0 | 0 | 0 | 1 | 0 | 0 | 10 | 70.00 |
| Dalblat | 0 | 0 | 0 | 0 | 4 | 0 | 0 | 0 | 0 | 0 | 0 | 0 | 0 | 0 | 0 | 0 | 0 | 2 | 0 | 0 | 0 | 0 | 0 | 0 | 6 | 66.67 |
| Ficurac | 0 | 0 | 0 | 0 | 0 | 10 | 0 | 1 | 0 | 0 | 0 | 0 | 0 | 0 | 1 | 0 | 0 | 0 | 0 | 0 | 0 | 0 | 0 | 0 | 12 | 83.33 |
| Ficurel | 0 | 0 | 0 | 0 | 0 | 0 | 6 | 0 | 0 | 0 | 0 | 0 | 0 | 0 | 0 | 0 | 0 | 0 | 0 | 0 | 0 | 0 | 0 | 0 | 6 | 100.00 |
| Ficuvir | 0 | 0 | 0 | 0 | 0 | 0 | 0 | 8 | 1 | 0 | 0 | 0 | 0 | 1 | 0 | 1 | 1 | 0 | 0 | 0 | 0 | 0 | 0 | 0 | 12 | 66.67 |
| Garupin | 0 | 0 | 0 | 0 | 0 | 0 | 0 | 0 | 2 | 0 | 0 | 0 | 0 | 0 | 1 | 0 | 0 | 0 | 0 | 0 | 0 | 0 | 0 | 0 | 3 | 66.67 |
| Haldcor | 0 | 0 | 0 | 0 | 0 | 0 | 0 | 0 | 1 | 9 | 0 | 0 | 1 | 0 | 0 | 0 | 0 | 0 | 0 | 0 | 0 | 0 | 0 | 0 | 11 | 81.82 |
| Lagelan | 0 | 0 | 0 | 0 | 0 | 0 | 2 | 1 | 0 | 0 | 5 | 1 | 0 | 0 | 0 | 0 | 0 | 0 | 0 | 0 | 0 | 0 | 0 | 0 | 9 | 55.56 |
| Lagepar | 0 | 0 | 0 | 0 | 0 | 0 | 0 | 0 | 0 | 0 | 0 | 4 | 0 | 0 | 0 | 0 | 0 | 0 | 0 | 0 | 0 | 0 | 0 | 0 | 4 | 100.00 |
| Madhlon | 0 | 0 | 0 | 0 | 0 | 0 | 0 | 0 | 1 | 0 | 0 | 0 | 19 | 1 | 0 | 0 | 1 | 0 | 0 | 0 | 0 | 0 | 0 | 1 | 23 | 82.61 |
| Mangind | 0 | 0 | 0 | 0 | 0 | 0 | 0 | 0 | 0 | 0 | 0 | 0 | 0 | 17 | 0 | 0 | 0 | 0 | 0 | 0 | 0 | 0 | 0 | 0 | 17 | 100.00 |
| Militom | 0 | 0 | 0 | 0 | 0 | 0 | 0 | 0 | 0 | 0 | 0 | 0 | 0 | 0 | 5 | 0 | 0 | 0 | 0 | 0 | 0 | 1 | 0 | 1 | 7 | 71.43 |
| Mitrpar | 0 | 0 | 0 | 1 | 0 | 0 | 0 | 1 | 0 | 0 | 0 | 0 | 0 | 0 | 0 | 5 | 1 | 0 | 0 | 0 | 0 | 0 | 2 | 0 | 10 | 50.00 |
| Others | 0 | 0 | 0 | 0 | 0 | 0 | 0 | 0 | 0 | 0 | 0 | 0 | 0 | 0 | 1 | 0 | 6 | 0 | 0 | 0 | 0 | 1 | 0 | 0 | 8 | 75.00 |
| Pongpin | 0 | 0 | 0 | 0 | 0 | 0 | 0 | 0 | 0 | 0 | 0 | 0 | 0 | 0 | 0 | 0 | 0 | 10 | 0 | 0 | 0 | 0 | 0 | 0 | 10 | 100.00 |
| Schlole | 0 | 0 | 0 | 0 | 0 | 1 | 2 | 0 | 0 | 0 | 0 | 1 | 1 | 0 | 0 | 0 | 0 | 0 | 6 | 0 | 0 | 0 | 0 | 0 | 11 | 54.55 |
| Syzycum | 0 | 0 | 0 | 0 | 0 | 0 | 0 | 0 | 0 | 0 | 0 | 0 | 0 | 0 | 0 | 0 | 0 | 0 | 0 | 5 | 0 | 0 | 0 | 0 | 5 | 100.00 |
| Tectgra | 1 | 0 | 0 | 0 | 0 | 0 | 0 | 0 | 0 | 0 | 0 | 0 | 0 | 0 | 0 | 0 | 0 | 0 | 0 | 0 | 12 | 0 | 0 | 0 | 13 | 92.31 |
| Termbel | 0 | 2 | 0 | 0 | 0 | 1 | 0 | 0 | 0 | 0 | 0 | 0 | 0 | 0 | 1 | 0 | 1 | 0 | 0 | 0 | 0 | 9 | 0 | 0 | 14 | 64.29 |
| Termtom | 0 | 1 | 0 | 0 | 0 | 0 | 0 | 0 | 0 | 0 | 0 | 0 | 0 | 0 | 0 | 0 | 0 | 0 | 0 | 0 | 0 | 0 | 12 | 0 | 13 | 92.31 |
| Wrigtin | 0 | 0 | 0 | 0 | 0 | 0 | 0 | 0 | 0 | 0 | 0 | 1 | 0 | 0 | 0 | 1 | 0 | 0 | 0 | 0 | 0 | 0 | 0 | 3 | 5 | 60.00 |
| Total | 8 | 18 | 7 | 9 | 4 | 12 | 10 | 11 | 5 | 9 | 6 | 7 | 21 | 19 | 11 | 7 | 12 | 13 | 6 | 5 | 13 | 12 | 14 | 8 |  |  |
| Producer's accuracy (%) | 87.50 | 66.67 | 100.00 | 77.78 | 100.00 | 83.33 | 60.00 | 72.73 | 40.00 | 100.00 | 83.33 | 57.14 | 90.48 | 89.47 | 45.45 | 71.43 | 50.00 | 76.92 | 100.00 | 100.00 | 92.31 | 75.00 | 85.71 | 37.50 |  |  |

| Table S5\| Error matrices for random forest classification of VNP. The top row of classes represents the reference trees, and the left column represents classified trees. | | | | | | | | | | | | | | | | | | | | | | | | | |
| --- | --- | --- | --- | --- | --- | --- | --- | --- | --- | --- | --- | --- | --- | --- | --- | --- | --- | --- | --- | --- | --- | --- | --- | --- | --- |
| Prediction | Albipro | Bambsps | Butemon | Dalblan | Dalblat | Diosmel | Ficurac | Ficurel | Garupin | Haldcor | Lagepar | Lanncor | Madhlon | Mangind | Militom | Mitrpar | Others | Ptermar | Tamaind | Tectgra | Termbel | Termtom | Wrigtin | Total | User's accuracy (%) |
| Albipro | 13 | 0 | 0 | 0 | 1 | 0 | 0 | 0 | 0 | 0 | 0 | 0 | 0 | 0 | 0 | 0 | 1 | 0 | 0 | 0 | 0 | 0 | 0 | 15 | 86.67 |
| Bambsps | 0 | 12 | 0 | 0 | 0 | 0 | 0 | 0 | 0 | 0 | 0 | 1 | 0 | 0 | 0 | 0 | 0 | 0 | 0 | 0 | 0 | 0 | 0 | 13 | 92.31 |
| Butemon | 0 | 0 | 8 | 0 | 0 | 0 | 0 | 0 | 0 | 0 | 0 | 0 | 0 | 0 | 0 | 0 | 0 | 0 | 0 | 2 | 0 | 0 | 0 | 10 | 80.00 |
| Dalblan | 0 | 0 | 0 | 8 | 0 | 0 | 0 | 0 | 0 | 0 | 0 | 0 | 0 | 0 | 0 | 0 | 0 | 0 | 0 | 0 | 0 | 0 | 0 | 8 | 100.00 |
| Dalblat | 0 | 0 | 0 | 0 | 4 | 0 | 0 | 0 | 0 | 0 | 0 | 0 | 0 | 0 | 0 | 0 | 1 | 0 | 0 | 0 | 0 | 0 | 0 | 5 | 80.00 |
| Diosmel | 0 | 0 | 1 | 0 | 0 | 3 | 0 | 0 | 0 | 1 | 0 | 0 | 0 | 0 | 0 | 0 | 0 | 0 | 0 | 0 | 0 | 0 | 0 | 5 | 60.00 |
| Ficurac | 0 | 0 | 0 | 0 | 0 | 1 | 7 | 0 | 0 | 0 | 0 | 0 | 0 | 0 | 0 | 0 | 0 | 0 | 0 | 0 | 0 | 0 | 0 | 8 | 87.50 |
| Ficurel | 0 | 0 | 0 | 0 | 0 | 0 | 0 | 11 | 0 | 1 | 0 | 0 | 0 | 0 | 0 | 0 | 0 | 0 | 0 | 0 | 0 | 0 | 0 | 12 | 91.67 |
| Garupin | 0 | 0 | 0 | 0 | 0 | 0 | 0 | 0 | 4 | 1 | 0 | 0 | 0 | 0 | 0 | 1 | 0 | 0 | 0 | 0 | 0 | 0 | 0 | 6 | 66.67 |
| Haldcor | 0 | 0 | 0 | 0 | 0 | 0 | 0 | 0 | 0 | 4 | 0 | 1 | 0 | 0 | 0 | 0 | 1 | 0 | 0 | 0 | 0 | 0 | 0 | 6 | 66.67 |
| Lagepar | 0 | 0 | 0 | 0 | 0 | 0 | 0 | 0 | 0 | 0 | 6 | 0 | 0 | 0 | 0 | 0 | 1 | 0 | 0 | 0 | 0 | 0 | 0 | 7 | 85.71 |
| Lanncor | 0 | 0 | 0 | 0 | 0 | 0 | 0 | 0 | 0 | 0 | 0 | 3 | 0 | 0 | 0 | 0 | 0 | 0 | 0 | 0 | 0 | 0 | 0 | 3 | 100.00 |
| Madhlon | 0 | 0 | 0 | 0 | 0 | 1 | 0 | 0 | 0 | 0 | 0 | 0 | 17 | 0 | 0 | 0 | 1 | 0 | 0 | 0 | 0 | 2 | 0 | 21 | 80.95 |
| Mangind | 0 | 0 | 0 | 0 | 0 | 0 | 1 | 0 | 0 | 0 | 0 | 0 | 0 | 12 | 0 | 0 | 0 | 0 | 0 | 0 | 0 | 0 | 0 | 13 | 92.31 |
| Militom | 0 | 0 | 0 | 0 | 0 | 0 | 0 | 0 | 0 | 0 | 0 | 0 | 0 | 0 | 5 | 0 | 0 | 0 | 0 | 0 | 0 | 0 | 0 | 5 | 100.00 |
| Mitrpar | 0 | 0 | 0 | 0 | 0 | 0 | 0 | 0 | 0 | 0 | 0 | 0 | 0 | 0 | 0 | 10 | 0 | 0 | 0 | 0 | 0 | 0 | 0 | 10 | 100.00 |
| Others | 0 | 0 | 0 | 0 | 0 | 0 | 0 | 0 | 0 | 0 | 0 | 0 | 0 | 0 | 0 | 0 | 5 | 1 | 0 | 0 | 0 | 0 | 0 | 6 | 83.33 |
| Ptermar | 0 | 0 | 0 | 0 | 1 | 0 | 0 | 0 | 2 | 0 | 0 | 1 | 0 | 0 | 0 | 0 | 1 | 3 | 0 | 2 | 0 | 0 | 0 | 10 | 30.00 |
| Tamaind | 0 | 0 | 0 | 0 | 0 | 0 | 2 | 0 | 0 | 0 | 0 | 0 | 0 | 0 | 0 | 0 | 0 | 0 | 7 | 0 | 0 | 0 | 0 | 9 | 77.78 |
| Tectgra | 0 | 0 | 0 | 0 | 0 | 0 | 0 | 0 | 0 | 0 | 0 | 0 | 0 | 0 | 0 | 0 | 0 | 0 | 0 | 6 | 0 | 0 | 0 | 6 | 100.00 |
| Termbel | 0 | 0 | 0 | 1 | 0 | 0 | 0 | 1 | 0 | 0 | 0 | 0 | 0 | 0 | 0 | 0 | 1 | 0 | 0 | 0 | 2 | 0 | 0 | 5 | 40.00 |
| Termtom | 0 | 0 | 1 | 0 | 0 | 0 | 0 | 0 | 1 | 1 | 0 | 0 | 0 | 0 | 0 | 0 | 1 | 0 | 0 | 0 | 0 | 16 | 0 | 20 | 80.00 |
| Wrigtin | 0 | 0 | 0 | 0 | 0 | 0 | 0 | 1 | 0 | 0 | 0 | 0 | 0 | 0 | 0 | 0 | 0 | 0 | 0 | 0 | 1 | 1 | 5 | 8 | 62.50 |
| Total | 13 | 12 | 10 | 9 | 6 | 5 | 10 | 13 | 7 | 8 | 6 | 6 | 17 | 12 | 5 | 11 | 13 | 4 | 7 | 10 | 3 | 19 | 5 |  |  |
| Producer's accuracy (%) | 100.00 | 100.00 | 80.00 | 88.89 | 66.67 | 60.00 | 70.00 | 84.62 | 57.14 | 50.00 | 100.00 | 50.00 | 100.00 | 100.00 | 100.00 | 90.91 | 38.46 | 75.00 | 100.00 | 60.00 | 66.67 | 84.21 | 100.00 |  |  |

| Table S6\| Error matrices for random forest classification of MTR. The top row of classes represents the reference trees, and the left column represents classified trees. | | | | | | | | | | | | | | | | | | | | | | | | |
| --- | --- | --- | --- | --- | --- | --- | --- | --- | --- | --- | --- | --- | --- | --- | --- | --- | --- | --- | --- | --- | --- | --- | --- | --- |
| Prediction | Anoglat | Artohet | Bambsps | Celttet | Dalblat | Eucaglo | Ficurac | Ficutsj | Ficuvir | Grevrob | Grewtil | Lagelan | Mangind | Others | Ptermar | Radexyl | Schlole | Stertet | Syzycum | Tectgra | Termbel | Termtom | Total | User's accuracy (%) |
| Anoglat | 10 | 0 | 0 | 0 | 0 | 0 | 0 | 0 | 0 | 0 | 0 | 0 | 0 | 0 | 1 | 0 | 0 | 0 | 0 | 0 | 2 | 0 | 13 | 71.43 |
| Artohet | 0 | 4 | 0 | 0 | 0 | 0 | 0 | 0 | 0 | 1 | 0 | 1 | 0 | 1 | 0 | 0 | 0 | 0 | 0 | 0 | 1 | 0 | 8 | 80.00 |
| Bambsps | 0 | 0 | 23 | 0 | 1 | 0 | 0 | 0 | 0 | 0 | 0 | 1 | 0 | 0 | 0 | 0 | 0 | 0 | 0 | 0 | 0 | 0 | 25 | 85.19 |
| Celttet | 0 | 0 | 0 | 3 | 0 | 0 | 0 | 0 | 0 | 0 | 0 | 0 | 0 | 0 | 0 | 0 | 0 | 0 | 0 | 0 | 0 | 0 | 3 | 100.00 |
| Dalblat | 0 | 0 | 0 | 0 | 19 | 0 | 1 | 0 | 0 | 0 | 0 | 0 | 0 | 0 | 2 | 0 | 1 | 0 | 1 | 0 | 1 | 0 | 25 | 76.00 |
| Eucaglo | 0 | 0 | 0 | 0 | 0 | 24 | 0 | 0 | 0 | 0 | 0 | 0 | 0 | 1 | 0 | 0 | 0 | 0 | 0 | 0 | 0 | 0 | 25 | 96.00 |
| Ficurac | 0 | 0 | 0 | 0 | 0 | 0 | 12 | 0 | 0 | 0 | 0 | 0 | 0 | 1 | 0 | 0 | 0 | 0 | 0 | 0 | 0 | 0 | 13 | 92.31 |
| Ficutsj | 0 | 0 | 0 | 0 | 0 | 0 | 0 | 4 | 0 | 0 | 0 | 0 | 0 | 0 | 0 | 0 | 0 | 0 | 0 | 0 | 0 | 0 | 4 | 100.00 |
| Ficuvir | 0 | 1 | 0 | 0 | 0 | 0 | 0 | 0 | 14 | 0 | 1 | 0 | 0 | 0 | 0 | 0 | 0 | 0 | 0 | 0 | 0 | 0 | 16 | 87.50 |
| Grevrob | 0 | 0 | 0 | 1 | 0 | 0 | 0 | 0 | 1 | 14 | 0 | 0 | 0 | 0 | 0 | 0 | 0 | 0 | 0 | 0 | 0 | 0 | 16 | 87.50 |
| Grewtil | 0 | 0 | 0 | 0 | 1 | 0 | 0 | 0 | 0 | 0 | 2 | 0 | 0 | 0 | 0 | 0 | 0 | 0 | 0 | 0 | 0 | 0 | 3 | 66.67 |
| Lagelan | 1 | 0 | 0 | 0 | 0 | 0 | 0 | 0 | 0 | 0 | 0 | 20 | 0 | 1 | 0 | 0 | 0 | 1 | 0 | 0 | 0 | 0 | 23 | 86.96 |
| Mangind | 0 | 0 | 0 | 0 | 0 | 0 | 0 | 0 | 0 | 0 | 0 | 0 | 7 | 0 | 0 | 0 | 0 | 0 | 0 | 0 | 0 | 0 | 7 | 100.00 |
| Others | 0 | 0 | 0 | 0 | 1 | 0 | 0 | 0 | 1 | 0 | 1 | 0 | 0 | 3 | 0 | 0 | 0 | 0 | 0 | 0 | 0 | 0 | 6 | 50.00 |
| Ptermar | 1 | 0 | 0 | 1 | 1 | 0 | 0 | 0 | 0 | 0 | 0 | 0 | 0 | 0 | 3 | 0 | 0 | 0 | 0 | 0 | 0 | 0 | 6 | 50.00 |
| Radexyl | 0 | 0 | 0 | 0 | 0 | 0 | 0 | 0 | 0 | 0 | 0 | 0 | 0 | 0 | 0 | 3 | 0 | 0 | 0 | 0 | 0 | 0 | 3 | 100.00 |
| Schlole | 0 | 0 | 0 | 0 | 0 | 0 | 0 | 0 | 0 | 0 | 0 | 0 | 0 | 0 | 0 | 0 | 12 | 0 | 1 | 0 | 0 | 0 | 13 | 92.31 |
| Stertet | 0 | 0 | 0 | 0 | 0 | 0 | 0 | 0 | 0 | 0 | 0 | 0 | 0 | 0 | 0 | 0 | 1 | 3 | 0 | 0 | 1 | 0 | 5 | 60.00 |
| Syzycum | 0 | 0 | 0 | 0 | 0 | 0 | 0 | 0 | 0 | 0 | 0 | 0 | 1 | 0 | 0 | 0 | 0 | 0 | 9 | 0 | 0 | 0 | 10 | 90.00 |
| Tectgra | 3 | 0 | 0 | 0 | 0 | 0 | 0 | 0 | 0 | 0 | 0 | 0 | 0 | 3 | 0 | 1 | 1 | 1 | 0 | 21 | 0 | 0 | 30 | 70.00 |
| Termbel | 1 | 0 | 0 | 0 | 0 | 0 | 0 | 0 | 0 | 0 | 0 | 0 | 0 | 2 | 0 | 0 | 1 | 0 | 0 | 0 | 8 | 2 | 14 | 57.14 |
| Termtom | 1 | 0 | 0 | 0 | 1 | 0 | 0 | 0 | 0 | 0 | 0 | 1 | 0 | 0 | 0 | 0 | 0 | 0 | 0 | 3 | 0 | 6 | 12 | 50.00 |
| Total | 17 | 5 | 23 | 5 | 24 | 24 | 13 | 4 | 16 | 15 | 4 | 23 | 8 | 12 | 6 | 4 | 16 | 5 | 11 | 24 | 13 | 8 |  |  |
| Producer's accuracy (%) | 58.82 | 80.00 | 100.00 | 60.00 | 79.17 | 100.00 | 92.31 | 100.00 | 87.50 | 93.33 | 50.00 | 86.96 | 87.50 | 25.00 | 50.00 | 75.00 | 75.00 | 60.00 | 81.82 | 87.50 | 61.54 | 75.00 |  |  |

| Table S7\| Error matrices for random forest classification of SRF. The top row of classes represents the reference trees, and the left column represents classified trees. | | | | | | | | | | | | | | | | | | | | | | | | | | | | |
| --- | --- | --- | --- | --- | --- | --- | --- | --- | --- | --- | --- | --- | --- | --- | --- | --- | --- | --- | --- | --- | --- | --- | --- | --- | --- | --- | --- | --- |
| Prediction | Aglamal | Albisam | Alstsch | Bambsps | Bombcei | Buchlan | Dalblat | Elaehyg | Elaetub | Eucaglo | Grewtil | Holiarn | Lagelan | Macapel | Mangind | Moricit | Ochltra | Others | Polyfra | Schlole | Stertet | Tectgra | Termbel | Termpan | Tetrnud | Xylixyl | Total | User's accuracy (%) |
| Aglamal | 8 | 0 | 0 | 0 | 0 | 0 | 0 | 0 | 0 | 0 | 0 | 0 | 0 | 1 | 0 | 0 | 0 | 2 | 1 | 0 | 0 | 0 | 0 | 0 | 0 | 0 | 12 | 66.67 |
| Albisam | 0 | 12 | 0 | 0 | 0 | 0 | 0 | 0 | 0 | 0 | 0 | 0 | 0 | 0 | 0 | 0 | 0 | 1 | 0 | 0 | 0 | 0 | 0 | 0 | 0 | 0 | 13 | 92.31 |
| Alstsch | 0 | 0 | 11 | 0 | 2 | 0 | 0 | 0 | 0 | 0 | 0 | 0 | 0 | 0 | 0 | 0 | 0 | 0 | 0 | 0 | 1 | 0 | 0 | 0 | 0 | 0 | 14 | 78.57 |
| Bambsps | 0 | 0 | 0 | 19 | 0 | 0 | 0 | 0 | 0 | 0 | 0 | 0 | 0 | 0 | 0 | 0 | 0 | 0 | 0 | 0 | 0 | 0 | 0 | 0 | 1 | 0 | 20 | 95.00 |
| Bombcei | 0 | 0 | 0 | 0 | 11 | 0 | 0 | 0 | 0 | 0 | 0 | 0 | 0 | 1 | 0 | 0 | 0 | 0 | 0 | 1 | 0 | 0 | 0 | 0 | 0 | 0 | 13 | 84.62 |
| Buchlan | 0 | 0 | 0 | 0 | 1 | 3 | 0 | 0 | 0 | 0 | 0 | 0 | 0 | 0 | 0 | 0 | 0 | 1 | 0 | 0 | 0 | 0 | 0 | 0 | 0 | 0 | 5 | 60.00 |
| Dalblat | 0 | 0 | 0 | 0 | 0 | 0 | 5 | 0 | 1 | 0 | 0 | 0 | 0 | 0 | 0 | 0 | 0 | 0 | 0 | 0 | 0 | 0 | 0 | 0 | 0 | 0 | 6 | 83.33 |
| Elaehyg | 0 | 0 | 0 | 0 | 0 | 1 | 1 | 2 | 1 | 0 | 0 | 0 | 0 | 0 | 0 | 0 | 0 | 0 | 0 | 1 | 0 | 0 | 1 | 0 | 0 | 0 | 7 | 28.57 |
| Elaetub | 0 | 0 | 0 | 0 | 0 | 0 | 0 | 0 | 4 | 0 | 0 | 0 | 0 | 0 | 0 | 0 | 0 | 1 | 1 | 0 | 0 | 0 | 1 | 0 | 0 | 0 | 7 | 57.14 |
| Eucaglo | 0 | 0 | 0 | 0 | 0 | 0 | 0 | 0 | 0 | 11 | 0 | 0 | 0 | 1 | 0 | 0 | 0 | 0 | 0 | 0 | 0 | 0 | 0 | 0 | 0 | 0 | 12 | 91.67 |
| Grewtil | 0 | 0 | 0 | 0 | 0 | 0 | 0 | 0 | 0 | 0 | 7 | 0 | 1 | 0 | 0 | 0 | 0 | 0 | 1 | 1 | 0 | 0 | 0 | 0 | 0 | 0 | 10 | 70.00 |
| Holiarn | 0 | 0 | 0 | 0 | 0 | 0 | 0 | 0 | 0 | 0 | 0 | 5 | 0 | 0 | 0 | 0 | 0 | 0 | 0 | 0 | 0 | 1 | 0 | 0 | 0 | 0 | 6 | 83.33 |
| Lagelan | 0 | 0 | 0 | 0 | 0 | 0 | 0 | 0 | 0 | 0 | 0 | 0 | 10 | 0 | 0 | 0 | 0 | 0 | 0 | 0 | 0 | 0 | 0 | 0 | 0 | 0 | 10 | 100.00 |
| Macapel | 0 | 1 | 0 | 0 | 0 | 0 | 0 | 1 | 0 | 0 | 0 | 0 | 1 | 5 | 0 | 1 | 0 | 2 | 0 | 0 | 0 | 4 | 0 | 1 | 0 | 0 | 16 | 31.25 |
| Mangind | 0 | 0 | 1 | 0 | 1 | 0 | 0 | 0 | 0 | 0 | 0 | 0 | 0 | 1 | 9 | 0 | 0 | 0 | 0 | 0 | 0 | 0 | 0 | 0 | 0 | 0 | 12 | 75.00 |
| Moricit | 0 | 0 | 1 | 0 | 0 | 0 | 0 | 0 | 0 | 0 | 0 | 0 | 0 | 0 | 0 | 10 | 0 | 0 | 0 | 0 | 0 | 0 | 0 | 0 | 0 | 0 | 11 | 90.91 |
| Ochltra | 0 | 0 | 0 | 0 | 0 | 0 | 0 | 0 | 0 | 0 | 0 | 0 | 0 | 0 | 0 | 0 | 6 | 0 | 0 | 0 | 0 | 0 | 0 | 0 | 0 | 0 | 6 | 100.00 |
| Others | 0 | 0 | 0 | 0 | 0 | 0 | 0 | 0 | 0 | 0 | 0 | 0 | 0 | 0 | 0 | 0 | 0 | 6 | 0 | 0 | 0 | 0 | 0 | 0 | 0 | 0 | 6 | 100.00 |
| Polyfra | 0 | 0 | 0 | 0 | 0 | 0 | 1 | 0 | 1 | 0 | 0 | 0 | 0 | 0 | 0 | 0 | 0 | 0 | 10 | 0 | 0 | 0 | 0 | 0 | 0 | 0 | 12 | 83.33 |
| Schlole | 0 | 0 | 0 | 0 | 1 | 0 | 0 | 0 | 0 | 0 | 0 | 0 | 0 | 0 | 0 | 0 | 0 | 0 | 0 | 11 | 1 | 1 | 0 | 0 | 0 | 0 | 14 | 78.57 |
| Stertet | 2 | 0 | 0 | 0 | 0 | 0 | 0 | 0 | 1 | 0 | 0 | 0 | 0 | 0 | 0 | 0 | 0 | 0 | 0 | 0 | 4 | 0 | 0 | 1 | 1 | 0 | 9 | 44.44 |
| Tectgra | 0 | 0 | 0 | 0 | 0 | 0 | 0 | 0 | 0 | 0 | 0 | 0 | 2 | 0 | 0 | 0 | 0 | 0 | 0 | 1 | 0 | 13 | 0 | 0 | 0 | 0 | 16 | 81.25 |
| Termbel | 0 | 0 | 0 | 0 | 0 | 0 | 0 | 0 | 0 | 0 | 0 | 0 | 0 | 0 | 0 | 0 | 0 | 0 | 0 | 0 | 0 | 0 | 5 | 0 | 0 | 1 | 6 | 83.33 |
| Termpan | 1 | 0 | 0 | 0 | 0 | 0 | 0 | 0 | 0 | 0 | 0 | 0 | 0 | 0 | 0 | 0 | 1 | 0 | 0 | 0 | 0 | 0 | 0 | 5 | 0 | 0 | 7 | 71.43 |
| Tetrnud | 0 | 0 | 0 | 0 | 0 | 1 | 0 | 0 | 0 | 0 | 0 | 0 | 0 | 1 | 0 | 0 | 0 | 0 | 0 | 0 | 0 | 0 | 0 | 0 | 9 | 0 | 11 | 81.82 |
| Xylixyl | 0 | 0 | 0 | 0 | 0 | 0 | 0 | 0 | 0 | 0 | 0 | 0 | 0 | 0 | 0 | 0 | 0 | 0 | 0 | 0 | 0 | 0 | 0 | 1 | 0 | 7 | 8 | 87.50 |
| Total | 11 | 13 | 13 | 19 | 16 | 5 | 7 | 3 | 8 | 11 | 7 | 5 | 14 | 10 | 9 | 11 | 7 | 13 | 13 | 15 | 6 | 19 | 7 | 8 | 11 | 8 |  |  |
| Producer's accuracy (%) | 72.73 | 92.31 | 84.62 | 100.00 | 68.75 | 60.00 | 71.43 | 66.67 | 50.00 | 100.00 | 100.00 | 100.00 | 71.43 | 50.00 | 100.00 | 90.91 | 85.71 | 46.15 | 76.92 | 73.33 | 66.67 | 68.42 | 71.43 | 62.50 | 81.82 | 87.50 |  |  |

| Table S8\| Mean values of functional diversity indices of tree communities at 0.005^⁰^ grid cell of the four species assemblage types. | | | | | |
| --- | --- | --- | --- | --- | --- |
| **PAs** | **Species assemblage types** | **Biophysical parameter** | | **Biochemical parameter** | |
|  |  | **FRic** | **FDiv** | **FRic** | **FDiv** |
| SWS | 1 | 15.31 | 0.74 | 6.23 | 0.78 |
| VNP | 1 | 20.13 | 0.67 | 7.20 | 0.76 |
|  | 4 | 17.82 | 0.68 | 7.07 | 0.79 |
| MTR | 2 | 15.37 | 0.76 | 5.71 | 0.65 |
|  | 4 | 15.55 | 0.79 | 5.91 | 0.84 |
| SRF | 2 | 8.99 | 0.77 | 4.63 | 0.90 |
|  | 3 | 11.92 | 0.81 | 5.85 | 0.76 |

| Table S9\| Number of plots in which a species is falling in DD at drier PAs. | | |
| --- | --- | --- |
| No. | **Species** | **DD plots** |
| 1 | Butemon | 256 |
| 2 | Mangind | 209 |
| 3 | Madhlon | 190 |
| 4 | Ficurel | 174 |
| 5 | Albipro | 162 |
| 6 | Dalblat | 161 |
| 7 | Pongpin | 144 |
| 8 | Ficurac | 143 |
| 9 | Termbel | 136 |
| 10 | Lagepar | 123 |
| 11 | Syzycum | 112 |
| 12 | Diosmel | 107 |
| 13 | Militom | 99 |
| 14 | Tamaind | 99 |
| 15 | Termtom | 96 |
| 16 | Wrigtin | 89 |
| 17 | Tectgra | 88 |
| 18 | Bambsps | 84 |
| 19 | Ficuvir | 74 |
| 20 | Haldcor | 73 |
| 21 | Garupin | 69 |
| 22 | Lagelan | 69 |
| 23 | Others | 63 |
| 24 | Lanncor | 47 |
| 25 | Bridret | 40 |
| 26 | Mitrpar | 39 |
| 27 | Schlole | 34 |
| 28 | Dalblan | 20 |
| 29 | Ptermar | 10 |

| Table S10\| Number of plots in which a species is falling in DD at wetter PAs. | | |
| --- | --- | --- |
| No. | **Species** | **DD plots** |
| 1 | Bambsps | 912 |
| 2 | Radexyl | 714 |
| 3 | Eucaglo | 708 |
| 4 | Stertet | 603 |
| 5 | Ptermar | 568 |
| 6 | Grewtil | 543 |
| 7 | Mangind | 484 |
| 8 | Tectgra | 484 |
| 9 | Anoglat | 472 |
| 10 | Ficutsj | 451 |
| 11 | Celttet | 431 |
| 12 | Termbel | 367 |
| 13 | Lagelan | 358 |
| 14 | Schlole | 339 |
| 15 | Dalblat | 329 |
| 16 | Grevrob | 325 |
| 17 | Syzycum | 311 |
| 18 | Ochltra | 290 |
| 19 | Termtom | 263 |
| 20 | Artohet | 243 |
| 21 | Ficuvir | 219 |
| 22 | Others | 206 |
| 23 | Aglamal | 185 |
| 24 | Elaetub | 181 |
| 25 | Polyfra | 166 |
| 26 | Termpan | 159 |
| 27 | Holiarn | 155 |
| 28 | Ficurac | 137 |
| 29 | Xylixyl | 128 |
| 30 | Moricit | 125 |
| 31 | Albisam | 123 |
| 32 | Elaehyg | 106 |
| 33 | Tetrnud | 100 |
| 34 | Buchlan | 68 |
| 35 | Bombcei | 48 |
| 36 | Macapel | 37 |
| 37 | Alstsch | 34 |

| Table S11\| Mean, median and range of species falling in DD in 0.5 ha plots at each PA. | | | |
| --- | --- | --- | --- |
| PAs | **Mean** | **Median** | **Range** |
| SWS | 4.09 | 4 | 1 - 12 |
| VNP | 3.38 | 3 | 1 - 15 |
| MTR | 5.41 | 5 | 1 - 15 |
| SRF | 4.8 | 4 | 1 - 17 |

| Table S12\| Plot numbers where a common abundant absent species falls in DD at each PA. | | | | |
| --- | --- | --- | --- | --- |
| Species | **SWS** | **VNP** | **MTR** | **SRF** |
| Bambsps | 52 | 32 | 782 | 130 |
| Dalblat | 142 | 19 | 177 | 152 |
| Mangind | 178 | 31 | 372 | 112 |
| Tectgra | 83 | 5 | 351 | 133 |
| Termbel | 56 | 80 | 248 | 119 |

| Table S13\| Species falling in DD at maximum number of plots along with their occurrence at four PAs. | | | |
| --- | --- | --- | --- |
| No. | **Species** | **DD plots** | **Species category** |
| 1 | Bambsps | 996 | Common in 4 PAs |
| 2 | Radexyl | 714 | PA-specific in MTR |
| 3 | Eucaglo | 708 | Common in MTR & SRF |
| 4 | Mangind | 693 | Common in 4 PAs |
| 5 | Stertet | 603 | Common in MTR & SRF |
| 6 | Ptermar | 578 | Common in VNP & MTR |
| 7 | Tectgra | 572 | Common in 4 PAs |
| 8 | Grewtil | 543 | Common in MTR & SRF |
| 9 | Termbel | 503 | Common in 4 PAs |
| 10 | Dalblat | 490 | Common in 4 PAs |
| 11 | Anoglat | 472 | PA-specific in MTR |
| 12 | Ficutsj | 451 | PA-specific in MTR |

| Table S14\| Location, topography, and decadal mean of rainfall and temperature of four PAs. | | | | | |
| --- | --- | --- | --- | --- | --- |
| PAs | **Location** | | **Average Elevation**  **(m)** | **Rainfall (mm)** | **Temperature (^°^C)** |
|  | **Latitude (^°^N)** | **Longitude (^°^E)** |  |  |  |
| SWS | 21.88 | 73.65 | 287 | 1140.13 | 27.08 |
| VNP | 20.82 | 73.44 | 169 | 1511.38 | 26.58 |
| MTR | 11.73 | 76.46 | 1233 | 1636.98 | 24.93 |
| SRF | 10.51 | 76.58 | 553 | 2757.60 | 28.08 |

Field data generation and processing

Extraction of abundant tree species

AVIRIS-NG data collection and processing

Extraction of canopy level spectral signatures of the abundant tree species

Developing abundant tree species maps using Random forest model

CHIRPS grids layout (0.5^⁰^ 0.005^⁰^ resolution)

0.5 ha plots data extraction from each of 0.005^⁰^ grid

Distribution details of abundant species at plots level

Extraction of grids level climate components and additional parameters

Testing this species distribution data to estimate dark diversity

Utilizing these two datasets to run SCGLR model

CA and Hierarchical clustering for cross-validation

Extrapolation of the model to the entire forest cover of each PA

Functional diversity estimates

Hierarchical clustering species assemblages

Cross-validation of CWMs and development of functional maps

**Figure. S1| Framework of the methodology.**

| Table S15\| Climatic components considered in this study. | | |
| --- | --- | --- |
| Code | **Description** | **Mean (range)** |
| C1 | Decadal Mean Temperature (^⁰^C) | 26.21 (24.57 ─ 28.35) |
| C5 | Max temp of warmest month (^⁰^C) | 29.66 (27.12 ─ 32.92) |
| C6 | Min temp of coldest month (^⁰^C) | 23.19 (21.19 ─ 24.46) |
| C12 | Decadal Mean Rainfall (mm) | 1831.37 (1126.25 ─ 3012.97) |
| C13 | Rainfall of wettest month (mm) | 422.92 (249.33 ─ 653.31) |
| C16 | Rainfall of wettest quarter (mm) | 912.28 (498.12 ─ 1370.90) |

| Table S16\| Flight details of AVIRIS-NG data. | | | | | | |
| --- | --- | --- | --- | --- | --- | --- |
| PAs | **Flight**  **Elevation**  **(km)** | **Image acquisition date** | **Image Acquisition Time (GMT +5:30)** | **Flight**  **footprint length (km)** | **Geographical area (sq. km)** | **Cloud cover** |
| MTR | 4.83 | 05-Jan-16 | 10:62:47 – 12:81:39 | 42.22 | 540.70 | Clear to hazy |
| SRF | 4.05 | 06-Jan-16 | 11:03:00 – 12:42:05 | 44.80 | 493.70 | Clear to cloudy |
| SWS | 4.15 | 08-Feb-16 | 12:58:43 – 13:61:58 | 44.85 | 501.88 | Clear |
| VNP | 4.16 | 09-Feb-16 | 11:02:17 – 11:57:46 | 13.32 | 82.36 | Clear |

| Table S17\| List of abundant species at four PAs. Values for Canopy Phenology of Evergreen (1) and Deciduous (0). | | | | |
| --- | --- | --- | --- | --- |
| Sr. No. | **Species** | **Family** | **Canopy Phenology** | **Code** |
| 1 | *Aglaia malabarica* | Meliaceae | Evergreen | Aglamal |
| 2 | *Albizia procera* | Fabaceae | Deciduous | Albipro |
| 3 | *Albizia saman* | Fabaceae | Deciduous | Albisam |
| 4 | *Alstonia scholaris* | Apocynaceae | Evergreen | Alstsch |
| 5 | *Anogeissus latifolia* | Combretaceae | Deciduous | Anoglat |
| 6 | *Artocarpus heterophyllus* | Moraceae | Evergreen | Artohet |
| 7 | Bamboo sps | Poaceae | Deciduous | Bambsps |
| 8 | *Bombax ceiba* | Malvaceae | Deciduous | Bombcei |
| 9 | *Bridelia retusa* | Phyllanthaceae | Deciduous | Bridret |
| 10 | *Buchanania lanceolata* | Anacardiaceae | Evergreen | Buchlan |
| 11 | *Butea monosperma* | Fabaceae | Deciduous | Butemon |
| 12 | *Celtis tetrandra* | Cannabaceae | Deciduous | Celttet |
| 13 | *Dalbergia lanceolaria* | Fabaceae | Deciduous | Dalblan |
| 14 | *Dalbergia latifolia* | Fabaceae | Deciduous | Dalblat |
| 15 | *Diospyros melanoxylon* | Ebenaceae | Deciduous | Diosmel |
| 16 | *Elaeocarpus hygrophilus* | Elaeocarpaceae | Evergreen | Elaehyg |
| 17 | *Elaeocarpus tuberculatus* | Elaeocarpaceae | Evergreen | Elaetub |
| 18 | *Eucalyptus globulus* | Myrtaceae | Evergreen | Eucaglo |
| 19 | *Ficus racemosa* | Moraceae | Deciduous | Ficurac |
| 20 | *Ficus religiosa* | Moraceae | Evergreen | Ficurel |
| 21 | *Ficus tsjahela* | Moraceae | Evergreen | Ficutsj |
| 22 | *Ficus virens* | Moraceae | Deciduous | Ficuvir |
| 23 | *Garuga pinnata* | Burseraceae | Deciduous | Garupin |
| 24 | *Grevillea robusta* | Proteaceae | Deciduous | Grevrob |
| 25 | *Grewia tiliifolia* | Malvaceae | Deciduous | Grewtil |
| 26 | *Haldina cordifolia* | Rubiaceae | Deciduous | Haldcor |
| 27 | *Holigarna arnottiana* | Anacardiaceae | Evergreen | Holiarn |
| 28 | *Lagerstroemia lanceolata* | Lythraceae | Deciduous | Lagelan |
| 29 | *Lagerstroemia parviflora* | Lythraceae | Deciduous | Lagepar |
| 30 | *Lannea coromandelica* | Anacardiaceae | Deciduous | Lanncor |
| 31 | *Macaranga peltata* | Euphorbiaceae | Deciduous | Macapel |
| 32 | *Madhuca longifolia* | Sapotaceae | Deciduous | Madhlon |
| 33 | *Mangifera indica* | Anacardiaceae | Evergreen | Mangind |
| 34 | *Miliusa tomentosa* | Annonaceae | Deciduous | Militom |
| 35 | *Mitragyna parvifolia* | Rubiaceae | Deciduous | Mitrpar |
| 36 | *Morinda citrifolia* | Rubiaceae | Evergreen | Moricit |
| 37 | *Ochlandra travancorica* | Poaceae | Evergreen | Ochltra |
| 38 | *Polyalthia fragrans* | Annonaceae | Evergreen | Polyfra |
| 39 | *Pongamia pinnata* | Fabaceae | Evergreen | Pongpin |
| 40 | *Pterocarpus marsupium* | Fabaceae | Deciduous | Ptermar |
| 41 | *Radarmachera xylocarpa* | Bignoniaceae | Deciduous | Radaxyl |
| 42 | *Schleichera oleosa* | Sapindaceae | Deciduous | Schlole |
| 43 | *Stereospermum tetragonum* | Bignoniaceae | Deciduous | Stertet |
| 44 | *Syzygium cumini* | Myrtaceae | Evergreen | Syzycum |
| 45 | *Tamarindus indica* | Fabaceae | Deciduous | Tamaind |
| 46 | *Tectona grandis* | Lamiaceae | Deciduous | Tectgra |
| 47 | *Terminalia bellirica* | Combretaceae | Deciduous | Termbel |
| 48 | *Terminalia paniculata* | Combretaceae | Deciduous | Termpan |
| 49 | *Terminalia tomentosa* | Combretaceae | Deciduous | Termtom |
| 50 | *Tetrameles nudiflora* | Tetramelaceae | Deciduous | Tetrnud |
| 51 | *Wrightia tinctoria* | Apocynaceae | Deciduous | Wrigtin |
| 52 | *Xylia xylocarpa* | Fabaceae | Deciduous | Xylixyl |
| 53 | Others⃰ | ─ | ─ | Others |

⃰Others consist of four to six species at each PA spread over 8%─10% of forest cover.

| Table S18\| Formulae of the vegetation indices used in this study. Chlorophyll/Carotenoid Index (CCI), Normalized Difference Water Index (NDWI), Near-infrared reflectance of vegetation multiplied by incoming sunlight (NIRvP) | | |
| --- | --- | --- |
| Indices | **Formulae** | **References number** |
| CCI | $\frac{532-630 nm}{532+630 nm}$ | 15 |
| NIRvP | NIRv × PAR | 16 and 17 |
| NDWI | $\frac{857-1241 nm}{857+1241 nm};sNDWI=\frac{NDWI+1}{2}$ | 21 |

⃰References number given are found in references section of main text.
